# Post-marketing safety profile of dengue vaccines CYD-TDV and TAK-003: analysis of adverse event reports from a European database

**DOI:** 10.3389/fphar.2026.1789762

**Published:** 2026-02-20

**Authors:** Viviana Maria Gianguzzo, Michelangelo Rottura, Federica Maria Sacco, Matthew Gavino Donadu, Giuseppina Sanna, Natasha Irrera, Egidio Imbalzano, Vincenzo Arcoraci, Giuseppinella Melita, Ylenia Marino, Sara Manti, Giovanni Pallio

**Affiliations:** 1 Department of Chemical, Biological, Pharmaceutical and Environmental Sciences, University of Messina, Messina, Italy; 2 Department of Clinical and Experimental Medicine, University of Messina, Messina, Italy; 3 Department of Medicine, Surgery and Pharmacy, Scuola di Specializzazione in Farmacia Ospedaliera, University of Sassari, Sassari, Italy; 4 Department of Biomedical Sciences, University of Cagliari, Monserrato, Italy; 5 Department of Human Pathology of Adult and Childhood Gaetano Barresi, University of Messina, Messina, Italy; 6 Department of Biomedical and Dental Sciences and Morphological and Functional Imaging, University of Messina, Messina, Italy

**Keywords:** adverse drug reactions, CYD-TDV, dengue, pharmacovigilance, TAK-003, vaccines

## Abstract

**Introduction:**

Dengue is one of the major global public health burden, particularly in endemic regions. CYD-TDV and TAK-003 are the currently licensed live attenuated tetravalent dengue vaccines, differing in serostatus indication, age range, and immunogenic design. While efficacy has been demonstrated in clinical trials, post-marketing safety data are still limited, supporting the need for real-world pharmacovigilance analyses.

**Objective:**

This study characterize adverse reactions associated with CYD-TDV and TAK-003 reported in the European pharmacovigilance database and to compare their post-marketing safety profiles.

**Methods:**

A retrospective pharmacovigilance study was conducted using the EudraVigilance database. Individual Case Safety Reports (ICSRs) listing CYD-TDV or TAK-003 as suspected products were retrieved. Adverse drug reactions were coded using MedDRA® version 28.1 and classified by seriousness and outcome. Disproportionality analyses were performed using Reporting Odds Ratios (RORs) with 95% confidence intervals at the System Organ Class (SOCs) and Preferred Terms (PTs) levels. Sensitivity analyses included grouping clinically related PTs and restricting analyses to serious cases. Vaccine groups were compared using chi-square tests.

**Results:**

A total of 2,288 ICSRs were identified, including 1,768 (77.3%) related to TAK-003 and 520 (22.7%) to CYD-TDV. TAK-003-related reports mainly involved adults (49.8%) and females (57.9%). Although most ICSRs were classified as serious, serious reports were more frequent for CYD-TDV than for TAK-003 (81.1% vs. 76.4%; p = 0.04). Fatal outcomes were also more commonly reported for CYD-TDV (50.8% vs. 0.6%; p < 0.01). At the SOC level, TAK-003 showed lower disproportional reporting than CYD-TDV for infections and infestations (ROR = 0.07; 95% CI = 0.056–0.089), gastrointestinal disorders (ROR = 0.22; 95%CI = 0.18–0.27), general disorders and administration site conditions (ROR = 0.29; 95%CI = 0.23–0.36), nervous system disorders (ROR = 0.42; 95%CI = 0.34–0.51), cardiac disorders (ROR = 0.35; 95%CI = 0.23–0.51), and hepatobiliary disorders (ROR = 0.09; 95%CI = 0.04–0.19). Higher reporting for TAK-003 was observed for skin and subcutaneous tissue disorders (ROR = 1.98; 95%CI = 1.59–2.48).

**Conclusion:**

This real-world pharmacovigilance study suggests that TAK-003 is predominantly associated with non-serious, reactogenic adverse reactions, whereas CYD-TDV reports more frequently involve serious outcomes, likely reflecting differences in indications and epidemiological contexts. Continued post-marketing surveillance remains essential for both vaccines.

## 1 Introduction

Dengue is one of the most widespread mosquito-borne diseases, with estimates suggesting that between 284 and 528 million people are infected annually (Harapan et al., 2020). The World Health Organization (WHO) has classified dengue as one of the most significant viral mosquito-borne diseases, affecting over half of the global population, especially those residing in high-risk areas (Kularatne and Dalugama, 2022). The vectors responsible for dengue transmission are highly prevalent in urban environments, especially in tropical and subtropical regions where the disease is endemic (Liu et al., 2023). Dengue is also an increasing concern among international travelers and among migrants arriving from highly endemic countries (Riddell and Babiker, 2017; Masyeni et al., 2018; Halstead and Wilder-Smith, 2019).

Clinical manifestations range from mild febrile illness to severe complications such as dengue hemorrhagic fever (DHF) and dengue shock syndrome (DSS), conditions that may lead to fatal outcomes (Moraes et al., 2013). Disease severity is influenced by several factors, including viral serotype (DENV-1 to DENV-4), host immune status, and other determinants. The lack of specific antiviral therapies emphasizes the urgent need for effective preventive measures. Although vector control remains the primary approach to reducing transmission, it has proven insufficient to contain outbreaks, underscoring the need for an effective vaccine (Kant et al., 2025).

CYD-TDV, Sanofi Pasteur (Lyon, France), was the first dengue vaccine to be approved worldwide. It received initial authorization in 2015 in the Philippines and, in 2019, by the U.S. FDA, although it has not yet been approved by the European Medicines Agency (EMA) in Europe. CYD-TDV is a recombinant, live - attenuated, tetravalent dengue vaccine constructed using the yellow fever 17D vaccine strain (YFV-17D) backbone with chimeric prM/E RNA sequences derived from the four dengue virus serotypes (Tonto et al., 2025). The vaccine is administered in a three-dose schedule (0, 6, and 12 months) to individuals aged 9–45 years who are already seropositive for dengue (European Medicines Agency, 2025a). In seronegative individuals, vaccine-induced antibodies may promote antibody-dependent enhancement (ADE), increasing the risk of severe dengue (Yang et al., 2018). Its efficacy has been demonstrated in several endemic countries, including the phase 3 CYD15 trial (NCT01374516), which reported a vaccine efficacy (VE) of 60.8% against virologically confirmed symptomatic dengue, with higher protection observed against hospitalized and severe forms (Villar et al., 2015). By contrast, TAK-003, Takeda Vaccines (Cambridge, MA, United States), is a more recently developed live attenuated tetravalent vaccine designed using a DENV-2 backbone with recombinant components from the other serotypes, conferring immunity against all four dengue virus serotypes (Wilder-Smith, 2024). TAK-003 induces robust humoral and T-cell–mediated immune responses (Sáez-Llorens et al., 2025). A 5-year clinical trial involving over 20,000 children and adolescents demonstrated its effectiveness in preventing severe dengue and reducing hospitalizations (Tricou et al., 2024). The vaccine was first approved in Indonesia for individuals aged 6–45 years. It subsequently received marketing authorization in the European Union in 2023 for individuals aged 4 years and over, regardless of previous dengue infection.

Although both vaccines have demonstrated efficacy and favorable safety profiles in randomized clinical trials (Reynales et al., 2020; Tricou et al., 2020; 2023; Biswal et al., 2021; de Moraes et al., 2022; Sirivichayakul et al., 2022; Luhm et al., 2023), they differ substantially in their indications and target populations. CYD-TDV is restricted to seropositive individuals due to the risk of ADE in seronegative recipients, whereas TAK-003 is indicated for both seropositive and seronegative individuals and therefore has broader potential use, including among travelers from non-endemic regions. These differences make a direct comparison of their respective safety profiles particularly relevant.

Furthermore, despite robust evidence from clinical trials, there are still important pharmacovigilance considerations. Live attenuated vaccines require continuous monitoring to detect rare or unexpected adverse events, and real-world post-marketing experience for both products is still limited. The use of dengue vaccines in populations not represented in clinical trials may reveal different safety patterns. Currently, there is a lack of post-marketing safety data from real-world conditions for both vaccines, emphasizing the need for studies based on spontaneous reporting systems.

Therefore, the aim of this study was to describe adverse drug reactions (ADRs) associated with both dengue vaccines as reported in a European pharmacovigilance database and, secondarily, to compare their respective safety profiles.

## 2 Methods

### 2.1 Data source and selection process

A retrospective pharmacovigilance analysis was conducted using the EudraVigilance (EV) platform (public interface available at https://www.adrreports.eu; accessed on 07 January 2026). EV functions as the central repository for spontaneous reports of suspected ADRs submitted within the European Union. The system was established to support the collection, organization, and evaluation of safety information on ADRs. It contains reports for medicinal products that are already marketed as well as for those undergoing assessment in clinical trials. Several publications have underscored the relevance of EV as a valuable source of real-world evidence for post-marketing safety monitoring (Postigo et al., 2018; Pozsgai et al., 2022; Cicala et al., 2024).

### 2.2 Reports selection

Individual Case Safety Reports (ICSRs) listing CYD-TDV or TAK-003 as suspected medicinal products were retrieved from the EV database. The analysis included ICSRs for CYD-TDV reported from 1 January 2018 to 31 December 2025, and for TAK-003 from 1 January 2023 to 31 December 2025. Data extraction was performed on 07 January 2026. Cases linked to the literature sources were excluded.

### 2.3 Data characteristics

Data elements retrieved from each ICSR included: EV case identification number, report submission date, and qualification of the primary reporting source. Additional variables encompassed patient demographics (sex and age group), ADR characteristics (type of ADR, duration, outcome, and seriousness criteria), and characteristics of suspected and concomitant drugs (administration route, therapy duration, dosages, and therapeutic indication).

All ADRs were coded according to MedDRA® version 28.1. The MedDRA terminology is organized into a hierarchical five-level structure progressing from the most specific to the most aggregated term: Lowest Level Terms (LLTs), Preferred Terms (PTs), High-Level Terms (HLTs), High-Level Group Terms (HLGTs), and System Organ Classes (SOCs). This structure has been described extensively in previous research (Zhang et al., 2024).

ADRs were categorized as “serious” when they were associated with any of the following: death, life-threatening conditions, hospitalization or its prolongation, persistent or significant disability/incapacity, congenital anomalies/birth defects, or when judged medically significant by the reporter. ADR outcomes were categorized using standardized terminology based on the information provided in the ICSR, namely,: “recovered/resolved,” “recovering/resolving,” “recovered/resolved with sequelae,” “not recovered/not resolved,” “fatal,” and “unknown.”

All medicinal products were classified according to the Anatomical Therapeutic and Chemical (ATC) Classification System. When multiple ADRs within the same ICSR were associated with different outcomes, an overall case outcome was determined using the “Lower Level of Resolution” approach, as outlined in previous studies (Monaco et al., 2017).

### 2.4 Data analysis

A descriptive analysis was performed to evaluate patient demographic characteristics, report features, and ADRs. Categorical variables were summarized using absolute counts and percentages. Comparisons between the ICSR groups were conducted using two-sided Pearson chi-square tests, with a significance threshold set at p < 0.05.

A disproportionality assessment was carried out using Reporting Odds Ratios (RORs) with corresponding 95% confidence intervals to explore potential differences in ADR reporting patterns. This analysis was conducted at both the MedDRA® System Organ Class (SOC) and Preferred Term (PT) levels. A positive signal was defined by a lower 95% CI bound exceeding 1, while a negative association required an upper 95% CI bound below 1. Only ADRs reported in at least three ICSRs were eligible for disproportionality testing.

Preferred Terms referring to rash (“Rash macular”, “Rash papular”, and “Rash maculo-papular”) were grouped into “Cutaneous rash” to avoid dilution across closely related MedDRA terms, consistent with common pharmacovigilance aggregation practices (Winter C., 2011). Similarly, the Preferred Terms “Injection site erythema” and “Vaccination site erythema” were combined into the category “Injection site erythema”.

All statistical analyses were conducted using SPSS Version 28 (IBM Corp., Armonk, NY, United States).

## 3 Results

A total of 2,288 ICSRs reporting TAK-003 or CYD-TDV as suspected medicinal products were retrieved from the EV platform. Of these, 77.3% (n = 1,768) involved TAK-003, while 20.7% (n = 520) involved CYD-TDV.

The characteristics of ICSRs associated with TAK-003 (N = 1,768) and CYD-TDV (N = 520) were reported in Table 1. A significant difference in sex distribution was observed between the two vaccine groups (p < 0.01). TAK-003-related ICSRs showed a higher proportion of female cases (57.9%), whereas CYD-TDV-related reports demonstrated a more balanced sex distribution (male 41.2% and female 42.7%). Significant differences were also identified in age group distribution (p < 0.01). TAK-003-related ICSRs predominantly involved adult populations (≥18 years; 49.8%), while CYD-TDV-related reports more frequently concerned pediatric and adolescent populations, particularly individuals aged 12–17 years (32.3%).

**TABLE 1:** Characteristics of ICSRs for TAK-003 and CYD-TDV.

Characteristics	TAK-003 N 1,768 (%)	CYD-TDV N = 520 (%)	*p-value*
Sex			
Male	653 (36.93)	214 (41.15)	<0.01
Female	1,023 (57.86)	222 (42.69)	​
Unknown	92 (5.20)	84 (16.15)	​
Age categories (years)			
≤11	397 (22.45)	125 (24.04)	<0.01
12–17	301 (17.02)	168 (32.31)	​
≥18	880 (49.77)	79 (15.19)	​
Unknown	190 (10.75)	148 (28.46)	​
Seriousness			
Serious	1,350 (76.36)	422 (81.15)	0.04
Non-serious	418 (23.64)	98 (18.85)	​
Outcome			
Fatal	10 (0.57)	264 (50.77)	<0.01
Not recovered/Not resolved	258 (14.59)	16 (3.08)	<0.01
Recovered with sequelae	6 (0.34)	5 (0.96)	0.18
Recovering/Resolving	209 (11.82)	12 (2.31)	<0.01
Recovered/Resolved	867 (49.04)	125 (24.04)	<0.01
Unknown	418 (23.64)	98 (18.85)	​
Primary source qualification			
Healthcare professional	1,269 (71.78)	237 (45.58)	<0.01
Non-healthcare professional	499 (28.22)	283 (54.42)	​

The majority of ICSRs in both groups were classified as serious according to regulatory criteria; however, a significantly higher proportion of serious reports was observed for CYD-TDV compared with TAK-003 (81.1% vs. 76.4%; p = 0.04).

Marked differences were observed in reported clinical outcomes. Notably, fatal outcomes were significantly more frequently reported in CYD-TDV-related ICSRs compared with TAK-003-related reports (50.8% vs. 0.6%; p < 0.01). Conversely, TAK-003-related reports showed significantly higher proportions of outcomes classified as not recovered/not resolved, recovering/resolving, and recovered/resolved (all p < 0.01). No significant differences were observed for the outcome recovered with sequelae (p = 0.18). A substantial proportion of ICSRs in both groups had outcomes classified as unknown (23.6% for TAK-003% and 18.9% for CYD-TDV). Finally, the primary source qualification differed significantly between the two groups (p < 0.01). TAK-003-related ICSRs were predominantly reported by healthcare professionals (71.8%), whereas most CYD-TDV-related reports originated from non-healthcare professionals (54.4%) (Table 1).

The SOC level disproportionality analysis revealed relevant differences in the reporting profiles of TAK-003 and CYD-TDV. For the majority of SOCs, the ROR was below 1.0, indicating lower disproportional reporting for TAK-003 compared with CYD-TDV. Specifically, significant RORs with 95% confidence intervals entirely below unity were observed for infections and infestations (ROR 0.07; 95% CI 0.056–0.089), gastrointestinal disorders (ROR 0.22; 95% CI 0.18–0.27), general disorders and administration site conditions (ROR 0.29; 95% CI 0.23–0.36), nervous system disorders (ROR 0.42; 95% CI 0.34–0.51), cardiac disorders (ROR 0.35; 95% CI 0.23–0.51), and eye disorders (ROR 0.48; 95% CI 0.36–0.64).

Similarly, reduced disproportionality for TAK-003 was observed across several clinically relevant SOCs, including hepatobiliary (ROR 0.09; 95% CI 0.04–0.19), metabolism and nutrition (ROR 0.09; 95% CI 0.06–0.14), renal and urinary (ROR 0.12; 95% CI 0.06–0.23), and vascular disorders (ROR 0.13; 95% CI 0.10–0.17).

Conversely, a signal of disproportionate reporting in favor of TAK-003 was identified for skin and subcutaneous tissue disorders, which showed a statistically significant ROR above unity (ROR 1.98; 95% CI 1.59–2.48), suggesting a higher relative frequency of reporting compared with CYD-TDV. For musculoskeletal and connective tissue disorders, the ROR was close to unity, and the confidence interval included 1 (ROR 1.04; 95% CI 0.83–1.30), indicating no meaningful difference in reporting frequency between the two vaccines for this SOC (Table 2).

**TABLE 2:** MedDRA^®^ system organ class according to study group.

System organ class (SOC)	TAK-003 N = 1,768 (%)	CYD-TDV N = 520 (%)	Total N = 2,288	TAK-003 vs. CYD-TDV ROR [95%CI]
Blood and lymphatic system disorders	83 (4.69)	52 (10.00)	135 (5.90)	0.44 [0.31; 0.64]
Cardiac disorders	60 (3.39)	48 (9.23)	108 (4.72)	0.35 [0.23; 0.51]
Congenital, familial and genetic disorders	2 (0.11)	7 (1.35)	9 (0.39)	0.08 [0.02; 0.40]
Ear and labyrinth disorders	22 (1.24)	11 (2.12)	33 (1.44)	0.58 [0.28; 1.21]
Endocrine disorders	4 (0.23)	3 (0.58)	7 (0.31)	0.39 [0.09; 1.75]
Eye disorders	158 (8.94)	88 (16.92)	246 (10.75)	0.48 [0.36; 0.64]
Gastrointestinal disorders	282 (15.95)	242 (46.54)	524 (22.90)	0.22 [0.18; 0.27]
General disorders and administration site conditions	821 (46.44)	390 (75.00)	1,211 (52.93)	0.29 [0.23; 0.36]
Hepatobiliary disorders	8 (0.45)	26 (5.00)	34 (1.49)	0.09 [0.04; 0.19]
Immune system disorders	187 (10.58)	122 (23.46)	309 (13.51)	0.39 [0.30; 0.50]
Infections and infestations	199 (11.26)	334 (64.23)	533 (23.30)	0.07 [0.06; 0.09]
Injury, poisoning and procedural complications	157 (8.88)	67 (12.88)	224 (9.79)	0.66 [0.49; 0.89]
Investigations	87 (4.92)	135 (25.96)	222 (9.70)	0.15 [0.11; 0.20]
Metabolism and nutrition disorders	27 (1.53)	76 (14.62)	103 (4.50)	0.09 [0.06; 0.14]
Musculoskeletal and connective tissue disorders	452 (25.57)	129 (24.81)	581 (25.39)	1.04 [0.83; 1.30]
Neoplasms benign, malignant and unspecified (incl cysts and polyps)	7 (0.40)	33 (6.35)	40 (1.75)	0.06 [0.03; 0.13]
Nervous system disorders	619 (35.01)	293 (56.35)	912 (39.86)	0.42 [0.34; 0.51]
Pregnancy, puerperium and perinatal conditions	8 (0.45)	9 (1.73)	17 (0.74)	0.26 [0.10; 0.67]
Product issues	3 (0.17)	0 (0)	3 (0.13)	—
Psychiatric disorders	48 (2.71)	69 (13.27)	117 (5.11)	0.18 [0.12; 0.27]
Renal and urinary disorders	13 (0.74)	31 (5.96)	44 (1.92)	0.12 [0.06; 0.23]
Reproductive system and breast disorders	20 (1.13)	14 (2.69)	34 (1.49)	0.41 [0.21; 0.82]
Respiratory, thoracic and mediastinal disorders	221 (12.50)	196 (37.69)	417 (18.23)	0.24 [0.19; 0.30]
Skin and subcutaneous tissue disorders	673 (38.07)	123 (23.65)	796 (34.79)	1.98 [1.59; 2.48]
Social circumstances	2 (0.11)	16 (3.08)	18 (0.79)	0.04 [0.01; 0.16]
Surgical and medical procedures	2 (0.11)	17 (3.27)	19 (0.83)	0.03 [0.01; 0.15]
Vascular disorders	98 (5.54)	164 (31.54)	262 (11.45)	0.13 [0.10; 0.17]

A total of 10,901 suspected adverse reactions were observed in all ICSRs, corresponding to a mean of 4.8 reactions per case. Notably, 6,106 suspected reactions were observed in the TAK-003 group, averaging at 3.5 reactions per ICSR, compared to 4,795 suspected reactions in the CYD-TDV group, with a mean of 9.2 reactions per ICSR.

The PT disproportionality analysis revealed a consistent pattern of disproportionate reporting in favor of TAK-003 compared to CYD-TDV for cutaneous and hypersensitivity-related events. The strongest association was identified for injection site erythema (ROR 10.44; 95% CI 3.30–33.06), followed by signals for pruritus (ROR 4.81; 95% CI 2.43–9.50), angioedema (ROR 3.74; 95% CI 1.62–8.65), urticaria (ROR 3.30; 95% CI 1.65–6.57), and rash-related PTs, including cutaneous rash (ROR 2.36; 95% CI 1.13–4.17) and rash pruritic (ROR 3.06; 95% CI 1.09–8.59). Disproportionate reporting was also observed for common reactogenicity-related PTs, including fatigue (ROR 2.48; 95% CI 1.63–3.77), myalgia (ROR 1.77; 95% CI 1.26–2.48), and arthralgia (ROR 1.62; 95% CI 1.12–2.34). Several additional PTs showed RORs above unity (e.g., influenza-like illness and limb discomfort), although some estimates were based on small numbers and wide confidence intervals. Conversely, multiple PTs indicative of more severe clinical presentations were reported less frequently in association with TAK-003 than with CYD-TDV, including dengue fever (ROR 0.05; 95% CI 0.03–0.06), and other infection- and complication-related PTs (e.g., pneumonia, sepsis), with confidence intervals consistently below 1 (Table 3).

**TABLE 3:** Preferred Terms with the highest and lowest RORs for TAK-003 compared with CYD-TDV.

Preferred terms (PTs)	TAK-003 N	CYD-TDV N	TAK-003 vs. CYD-TDV ROR [95%CI]
Pruritus	138	9	4.81 [2.43; 9.50]
Angioedema	74	6	3.74 [1.62; 8.65]
Urticaria	97	9	3.30 [1.65; 6.57]
Incorrect route of product administration	82	8	3.11 [1.50; 6.48]
Rash pruritic	41	4	3.06 [1.09; 8.59]
Tachycardia	28	3	2.77 [0.84; 9.16]
Paraesthesia	37	4	2.76 [0.98; 7.77]
Fatigue	204	26	2.48 [1.63; 3.77]
Erythema	56	7	2.40 [1.09; 5.29]
Vaccination site pain	44	6	2.19 [0.93; 5.16]
Myalgia	243	43	1.77 [1.26; 2.48]
Arthralgia	190	36	1.62 [1.12; 2.34]
Hyperhidrosis	21	4	1.55 [0.53; 4.54]
Chills	95	19	1.50 [0.91; 2.48]
Syncope	97	20	1.45 [0.89; 2.37]
Illness	4	53	0.02 [0.01; 0.06]
Dengue fever	59	222	0.05 [0.03; 0.06]
Pneumonia	9	45	0.05 [0.03; 0.11]
Platelet count decreased	5	24	0.06 [0.02; 0.15]
Haematemesis	4	19	0.06 [0.02; 0.18]
Swelling	6	27	0.06 [0.03; 0.15]
Loss of consciousness	11	39	0.08 [0.04; 0.15]
Abdominal distension	4	14	0.08 [0.03; 0.25]
Vaccination failure	13	42	0.08 [0.04; 0.16]
Gastroenteritis	3	10	0.09 [0.02; 0.32]
Restlessness	3	10	0.09 [0.02; 0.32]
Sepsis	4	13	0.09 [0.03; 0.27]
Decreased appetite	17	51	0.09 [0.05; 0.16]
Epistaxis	9	28	0.09 [0.04; 0.19]
Gait disturbance	8	24	0.09 [0.04; 0.21]

The analysis of ICSRs identified several PTs that were reported exclusively in association with CYD-TDV and were not observed in ICSR related to TAK-003. These PTs primarily comprised serious neurological, hemorrhagic, and multi-system conditions. Among the most frequently reported were cerebral and internal hemorrhage, noninfective encephalitis, brain oedema, and pulmonary or visceral hemorrhage. Other clinically relevant PTs included multiple organ dysfunction syndrome, coma, septic shock, disseminated intravascular coagulation, and myocardial hemorrhage. While the presence of these PTs does not imply a causal relationship, their exclusive reporting in CYD-TDV ICSR suggests that they warrant further monitoring and careful evaluation in post-marketing safety assessments.

PTs reported exclusively in TAK-003 ICSR were also identified and are presented in the Supplementary Material. However, this analysis focused on PTs uniquely associated with CYD-TDV due to their frequency and potential clinical relevance.

The complete list of PTs reported exclusively for CYD-TDV or for TAK-003 is provided in the Supplementary Material (Supplementary Table S1).

## 4 Discussion

In this pharmacovigilance study based on EV data, we compared the post-marketing safety profiles of the two currently available dengue vaccines, TAK-003 and CYD-TDV. Overall, our findings highlight substantial differences in reporting patterns, seriousness, clinical outcomes, and adverse reaction profiles between the two vaccines, likely reflecting not only intrinsic differences in vaccine design but also distinct target populations, indications, and historical contexts of use.

TAK-003 accounted for more than three-quarters of all dengue vaccine–related ICSRs retrieved from the EV database. This predominance is plausibly explained by its more recent authorization in the European Union and its broader indication irrespective of baseline dengue serostatus, in contrast to CYD-TDV, whose use is restricted to seropositive individuals due to the risk of ADE in seronegative recipients (Sridhar et al., 2018; Author anonymous, 2019). Differences in age distribution, baseline dengue serostatus, and target populations are likely to play a central role in shaping the observed reporting patterns between CYD-TDV and TAK-003. Thus, CYD-TDV-related ICSRs predominantly concerned pediatric and adolescent populations, reflecting its historical use in dengue-endemic settings and its restriction to seropositive individual (Hadinegoro et al., 2015; Godói et al., 2017). In contrast, TAK-003-related reports mainly involved adults and individuals vaccinated irrespective of baseline serostatus, consistent with its broader indication and more recent use, including in non-endemic or travel-related contexts. These substantial differences in target populations and vaccination strategies should be taken into account when interpreting comparative pharmacovigilance findings.

A higher proportion of female cases was also observed among ICSRs related to TAK-003, indicating sex-related differences. Such findings are commonly reported in spontaneous reporting systems and may reflect sex-related differences in immune response, healthcare-seeking behavior, or reporting practices rather than a true difference in vaccine-related risk (Klein and Flanagan, 2016).

In terms of severity, most ICSRs for both vaccines were classified as serious according to regulatory criteria; notably, a significantly higher proportion of serious reports was observed for CYD-TDV.

Importantly, results derived from spontaneous reporting systems such as EudraVigilance should be interpreted with caution, as they primarily reflect reporting behaviors rather than causal associations. In this context, the higher proportion of serious and fatal outcomes reported in CYD-TDV-related ICSRs should not be interpreted as evidence of increased vaccine-related risk. Instead, these findings are more plausibly explained by confounding by indication, differences in epidemiological settings, baseline disease severity, and reporting bias, all of which are well-recognized limitations of pharmacovigilance databases.

Moreover, CYD-TDV has been available on the market for longer and has been subject to enhanced surveillance due to concerns regarding severe dengue and ADE in specific populations (Hadinegoro et al., 2015; Wilder-Smith et al., 2019). Moreover, CYD-TDV has primarily been deployed in dengue-endemic areas characterized by high background morbidity and mortality, which may have influenced the clinical context and reporting patterns of serious outcomes observed in spontaneous reports. TAK-003 accounted for more than three-quarters of all dengue vaccine–related ICSRsThe analysis of clinical outcomes revealed marked differences between the two vaccines. Fatal outcomes were reported substantially more frequently in CYD-TDV-related ICSRs, whereas TAK-003-related reports more commonly described non-fatal outcomes, including recovered/resolved or recovering/resolving cases. In addition, long-term follow-up of CYD-TDV clinical trials has shown an increased risk of severe dengue in seronegative individuals, which led to revised recommendations and restricted use (Hadinegoro et al., 2015; Sridhar et al., 2018).

Disproportionality analyses at both SOC and PT levels further highlighted a distinct reporting profile for TAK-003. Most SOCs showed significantly lower reporting odds for TAK-003 compared with CYD-TDV, particularly for infections, nervous system and cardiovascular disorders, and dengue-related conditions. Conversely, TAK-003 showed higher disproportionality for skin and subcutaneous tissue disorders, including injection site erythema, pruritus, urticaria, angioedema, and rash-related terms. These findings are consistent with the reactogenicity profile observed in TAK-003 clinical trials, where local and mild systemic reactions were the most frequently reported adverse events (Biswal et al., 2019; Tricou et al., 2024; European Medicines Agency, 2025b).

At the PT level, increased reporting of fatigue, myalgia, and arthralgia was also observed for TAK-003. These events are well described in the Summary of Product Characteristics and in phase 2 and phase 3 trials of TAK-003 and are generally considered transient and self-limiting (Biswal et al., 2019; Tricou et al., 2024). A recent post-marketing signal of anaphylaxis associated with TAK-003 vaccination reported by Brazilian pharmacovigilance authorities further supports the importance of continued monitoring of hypersensitivity reactions, although regulatory measures were promptly implemented and no vaccine-related deaths were identified (Agência Nacional de Vigilância Sanitária. Brazil, 2024).

In addition, the comparison of vaccine-specific PTs showed that several PTs were exclusively reported in CYD-TDV-related ICSRs and were not observed in TAK-003 ICSRs. These PTs mainly included serious neurological, hemorrhagic, and multi-organ conditions, such as cerebral and internal hemorrhage, noninfective encephalitis, brain oedema, and multiple organ dysfunction syndrome. The exclusive reporting of serious neurological and haemorrhagic PTs in CYD-TDV-related ICSRs represents an important observation that requires careful contextualization. Dengue virus infection is well recognized to be associated with neurological involvement (e.g., encephalitis/encephalopathy and immune-mediated neuroinflammatory disorders) and haemorrhagic complications driven by thrombocytopenia, platelet dysfunction, and vasculopathy/endothelial dysfunction (Naderian et al., 2025). In this context, the occurrence of such events in spontaneous reports is likely influenced by the clinical severity of dengue disease and the epidemiological settings in which CYD-TDV has historically been used, rather than indicating a direct vaccine-related adverse effect. Therefore, while these PTs warrant continued post-marketing monitoring, their presence should be interpreted within the broader clinical and epidemiological background of dengue infection. Importantly, this pattern is likely influenced by differences in target populations, age distribution, baseline dengue serostatus, and epidemiological settings in which CYD-TDV has historically been used. These settings are often characterized by a higher baseline risk of severe dengue and related complications, as reported in systematic reviews and long-term safety follow-up studies (Godói et al., 2017; Forrat et al., 2021). In contrast, available clinical trial and meta-analytic evidence indicate that TAK-003 is predominantly associated with non-serious and reactogenic adverse events, with no consistent signals of severe outcomes identified to date (Flacco et al., 2024). The complete list of vaccine-specific PTs is provided in the Supplementary Material.

Several limitations inherent to spontaneous reporting systems must be acknowledged. These include under-reporting, differential reporting stimulated by media attention or regulatory actions, lack of exposure denominators, and incomplete clinical information (Hazell and Shakir, 2006; Pariente et al., 2010). Additionally, differences in reporting source qualification between TAK-003 and CYD-TDV may have influenced the type and severity of reported events.

Beyond epidemiological and population-related factors, recent evidence suggests that dengue virus infection is associated with profound immune modulation and metabolic reprogramming, involving alterations in glucose and lipid metabolism, mitochondrial function, and inflammatory signaling pathways. These immunometabolic changes have been shown to influence innate and adaptive immune responses, endothelial activation, and systemic inflammatory processes, all of which contribute to disease severity and multisystem involvement (Chermahini et al., 2025). While these mechanisms are primarily related to dengue virus pathophysiology rather than vaccine-induced effects, they provide a broader biological framework for interpreting severe clinical manifestations reported in pharmacovigilance databases. In this context, immunometabolic dysregulation may shape the clinical background in which adverse events are reported following dengue vaccination, particularly in high-risk populations, without implying a direct causal relationship with vaccination.

In conclusion, this real-world pharmacovigilance analysis indicates that TAK-003 is predominantly associated with non-serious, reactogenic, and hypersensitivity-related adverse reactions, whereas CYD-TDV reports more frequently involve serious and fatal outcomes, likely influenced by its restricted indication and historical context of use.

At SOCs level, TAK-003 demonstrated a lower rate of disproportional reporting across most clinically relevant SOCs. Meanwhile, a consistent signal emerged for skin and subcutaneous tissue disorders.

At PTs level, this signal was mainly driven by local and hypersensitivity-related reactions, including injection site reactions, pruritus, urticaria, angioedema, and rash-related terms, which were largely non-serious and self-limiting. In contrast, the exclusive reporting of certain PTs in CYD-TDV-related ICSRs should be interpreted in light of the differences in target populations, baseline risk profiles, and epidemiological settings discussed above, rather than as an indication of vaccine-related causality.

Overall, these findings are consistent with the available clinical and post-marketing evidence, supporting the favourable safety profile of TAK-003, while underscoring the need for ongoing post-marketing surveillance of both dengue vaccines (Biswal et al., 2019; Tricou et al., 2024; European Medicines Agency, 2025a).

## 5 Strengths and limitations

One of the key strengths of this study is its utilization of the EV database, which is among the largest spontaneous reporting systems worldwide. This allowed for the evaluation of real-world safety data for dengue vaccines across multiple countries and healthcare settings. The broad coverage of the database enhances the generalizability of the findings beyond the controlled conditions of clinical trials. The comparative design represents an additional strength. By directly comparing TAK-003 and CYD-TDV, the analysis was able to identify differential reporting patterns that may not emerge from single-product evaluations, providing a more informative context for signal interpretation. The combined use of descriptive statistics and disproportionality analyses at both SOC and PT levels further strengthened the assessment, enabling identification of coherent and biologically plausible safety patterns.

Finally, the consistency observed between post-marketing findings and ADRs reported in pre-authorization clinical trials reinforces the robustness and relevance of the results for regulatory pharmacovigilance activities.

This study is subject to the inherent limitations of spontaneous reporting systems, including under-reporting, reporting biases, and the absence of exposure denominators, which preclude reliable estimation of incidence rates and causal inference. Differences in reporting behavior related to time since market authorization, media attention, and regulatory actions may have influenced the observed reporting patterns. Moreover, EV is a regulatory pharmacovigilance database that includes reports originating from both European Economic Area and non-European countries and does not systematically capture information on the geographical location of dengue virus acquisition or the total number of vaccinated individuals. Consequently, regional epidemiological patterns and disease incidence within Europe cannot be reliably assessed using this data source.

In addition, the completeness and quality of ICSRs are variable, with frequent missing information on patient characteristics, clinical history, comorbidities, and outcomes, limiting the ability to adjust for potential confounding factors. Finally, differences indications, target populations, and vaccination contexts between TAK-003 and CYD-TDV should be taken into account when interpreting comparative safety findings.

## Data Availability

The datasets presented in this study can be found in online repositories. The names of the repository/repositories and accession number(s) can be found below: EudraVigilance platform, public interface available at https://www.adrreports.eu; accessed on 07 January 2026.

## References

[B2] Author anonymous (2019). Dengue vaccine WHO position paper, September 2018 – recommendations. Vaccine 37, 4848–4849. 10.1016/j.vaccine.2018.09.063 30424888

[B3] Biswal S. Reynales H. Saez-Llorens X. Lopez P. Borja-Tabora C. Kosalaraksa P. (2019). Efficacy of a tetravalent dengue vaccine in healthy children and adolescents. N. Engl. J. Med. 381, 2009–2019. 10.1056/NEJMoa1903869 31693803

[B4] Biswal S. Mendez Galvan J. F. Macias Parra M. Galan-Herrera J.-F. Carrascal Rodriguez M. B. Rodriguez Bueno E. P. (2021). Immunogenicity and safety of a tetravalent dengue vaccine in dengue-naïve adolescents in Mexico City. Rev. Panam. Salud Pública 45, 1. 10.26633/RPSP.2021.67 34131423 PMC8196333

[B5] Chermahini F. A. Arvejeh P. M. Marincola F. M. Ahmad S. Naderian R. Pajand O. (2025). Investigating how dengue virus-induced metabolic changes affect the host immune response and how to develop immunomodulatory strategies. Virol. J. 22, 117. 10.1186/s12985-025-02745-3 40281578 PMC12023479

[B6] Cicala G. Rottura M. Gianguzzo V. M. Cristiano F. Drago S. F. A. Pallio G. (2024). Safety of inclisiran: a disproportionality analysis from the EudraVigilance database. Pharmaceuticals 17, 1365. 10.3390/ph17101365 39459005 PMC11511047

[B7] de Moraes J. C. Riediger I. N. Crosewski F. Garrett D. O. Fantinato F. F. Ribeiro K. B. (2022). A case-control study to determine the effectiveness of a tetravalent dengue vaccine in the state of Paraná, Brazil. Lancet Regional Health - Am. 7, 100141. 10.1016/j.lana.2021.100141 36777647 PMC9904136

[B8] European Medicines Agency (2025a). Qdenga (TAK-003): summary of product characteristics. Available online at: https://www.ema.europa.eu/it/documents/product-information/qdenga-epar-product-information_it.pdf (Accessed January 07, 2026).

[B9] European Medicines Agency (2025b). Dengvaxia (CYD-TDV): summary of product characteristics. Available online at: https://www.ema.europa.eu/en/documents/product-information/dengvaxia-epar-product-information_en.pdf. (Accessed January 07, 2026).

[B10] Flacco M. E. Bianconi A. Cioni G. Fiore M. Calò G. L. Imperiali G. (2024). Immunogenicity, safety and efficacy of the dengue vaccine TAK-003: a meta-analysis. Vaccines (Basel) 12, 770. 10.3390/vaccines12070770 39066408 PMC11281463

[B11] Forrat R. Dayan G. H. DiazGranados C. A. Bonaparte M. Laot T. Capeding M. R. (2021). Analysis of hospitalized and severe dengue cases over the 6 years of Follow-up of the tetravalent dengue vaccine (CYD-TDV) efficacy trials in Asia and Latin America. Clin. Infect. Dis. 73, 1003–1012. 10.1093/cid/ciab288 33822015 PMC8442794

[B12] Godói I. P. Lemos L. L. P. de Araújo V. E. Bonoto B. C. Godman B. Guerra Júnior A. A. (2017). CYD-TDV dengue vaccine: systematic review and meta-analysis of efficacy, immunogenicity and safety. J. Comp. Eff. Res. 6, 165–180. 10.2217/cer-2016-0045 28084784

[B13] Hadinegoro S. R. Arredondo-García J. L. Capeding M. R. Deseda C. Chotpitayasunondh T. Dietze R. (2015). Efficacy and long-term safety of a dengue vaccine in regions of endemic disease. N. Engl. J. Med. 373, 1195–1206. 10.1056/NEJMoa1506223 26214039

[B14] Halstead S. Wilder-Smith A. (2019). Severe dengue in travellers: pathogenesis, risk and clinical management. J. Travel Med. 26. 10.1093/jtm/taz062 31423536

[B15] Harapan H. Michie A. Sasmono R. T. Imrie A. (2020). Dengue: a minireview. Viruses 12, 829. 10.3390/v12080829 32751561 PMC7472303

[B16] Hazell L. Shakir S. A. W. (2006). Under-reporting of adverse drug reactions. Drug Saf. 29, 385–396. 10.2165/00002018-200629050-00003 16689555

[B17] Kant R. Singh A. Ali A. B. Nagre S. Ganguly N. K. Rana R. (2025). Dengue vaccine effectiveness and safety: a systematic analysis of recent clinical trial data. Virol. J. 22, 384. 10.1186/s12985-025-02937-x 41287091 PMC12641897

[B18] Klein S. L. Flanagan K. L. (2016). Sex differences in immune responses. Nat. Rev. Immunol. 16, 626–638. 10.1038/nri.2016.90 27546235

[B19] Kularatne S. A. Dalugama C. (2022). Dengue infection: global importance, immunopathology and management. Clin. Med. 22, 9–13. 10.7861/clinmed.2021-0791 35078789 PMC8813012

[B20] Liu Z. Zhang Q. Li L. He J. Guo J. Wang Z. (2023). The effect of temperature on dengue virus transmission by aedes mosquitoes. Front. Cell. Infect. Microbiol. 13, 1242173. 10.3389/fcimb.2023.1242173 37808907 PMC10552155

[B21] Luhm K. R. Shimakura S. E. Raboni S. M. Vieira da Costa-Ribeiro M. C. Diaz-Quijano F. A. de Mello A. M. (2023). Dengue vaccine effectiveness: results from a six-year population-based cohort study in southern Brazil. 10.1101/2023.12.28.23300598 PMC12874932 41662753

[B22] Masyeni S. Yohan B. Somia I. K. A. Myint K. S. A. Sasmono R. T. (2018). Dengue infection in international travellers visiting Bali, Indonesia. J. Travel Med. 25. 10.1093/jtm/tay061 30113689 PMC6118167

[B23] Monaco L. Biagi C. Conti V. Melis M. Donati M. Venegoni M. (2017). Safety profile of the direct oral anticoagulants: an analysis of the WHO database of adverse drug reactions. Br. J. Clin. Pharmacol. 83, 1532–1543. 10.1111/bcp.13234 28071818 PMC5465343

[B24] Moraes G. H. de Fátima Duarte E. Duarte E. C. (2013). Determinants of mortality from severe dengue in Brazil: a population-based case-control study. Am. Soc. Trop. Med. Hyg. 88, 670–676. 10.4269/ajtmh.11-0774 23400577 PMC3617850

[B25] Naderian R. Paraandavaji E. Maddah A. H. Keshavarzi S. Habibian A. Naderian R. (2025). Pathophysiology and clinical implications of dengue-associated neurological disorders. Front. Microbiol. 16, 1536955. 10.3389/fmicb.2025.1536955 41019525 PMC12463822

[B26] Pariente A. Didailler M. Avillach P. Miremont‐Salamé G. Fourrier‐Reglat A. Haramburu F. (2010). A potential competition bias in the detection of safety signals from spontaneous reporting databases. Pharmacoepidemiol. Drug Saf. 19, 1166–1171. 10.1002/pds.2022 20848561

[B44] Percio J. Kobayashi C. D. Silva R. M. A. Marinho A. K. B. B. Capovilla L. Andrade P. H. S. (2024). Safety signal detected: Anaphylaxis after attenuated dengue vaccine (TAK‐003) – Brazil, march 1, 2023–march 11, 2024. Vaccine 42, 126407. 10.1016/j.vaccine.2024.126407 39368126

[B27] Postigo R. Brosch S. Slattery J. van Haren A. Dogné J.-M. Kurz X. (2018). EudraVigilance medicines safety database: publicly accessible data for research and public health protection. Drug Saf. 41, 665–675. 10.1007/s40264-018-0647-1 29520645 PMC5990579

[B28] Pozsgai K. Szűcs G. Kőnig-Péter A. Balázs O. Vajda P. Botz L. (2022). Analysis of pharmacovigilance databases for spontaneous reports of adverse drug reactions related to substandard and falsified medical products: a descriptive study. Front. Pharmacol. 13. 10.3389/fphar.2022.964399 36147337 PMC9485933

[B29] Reynales H. Carrasquilla G. Zambrano B. Cortés S M. Machabert T. Jing J. (2020). Secondary analysis of the efficacy and safety trial data of the tetravalent dengue vaccine in children and adolescents in Colombia. Pediatr. Infect. Dis. J. 39, e30–e36. 10.1097/INF.0000000000002580 32040014 PMC7182239

[B30] Riddell A. Babiker Z. O. E. (2017). Imported dengue fever in east London: a 6-year retrospective observational study. J. Travel Med. 24. 10.1093/jtm/tax015 28355620

[B31] Sáez-Llorens X. DeAntonio R. Low J. G. H. Kosalaraksa P. Dean H. Sharma M. (2025). TAK-003: development of a tetravalent dengue vaccine. Expert Rev. Vaccines 24, 324–338. 10.1080/14760584.2025.2490295 40207772

[B32] Sirivichayakul C. Barranco-Santana E. A. Rivera I. E. Kilbury J. Raanan M. Borkowski A. (2022). Long-term safety and immunogenicity of a tetravalent dengue vaccine candidate in children and adults: a randomized, placebo-controlled, phase 2 study. J. Infect. Dis. 225, 1513–1520. 10.1093/infdis/jiaa406 32658250 PMC9071315

[B33] Sridhar S. Luedtke A. Langevin E. Zhu M. Bonaparte M. Machabert T. (2018). Effect of dengue serostatus on Dengue vaccine safety and efficacy. N. Engl. J. Med. 379, 327–340. 10.1056/NEJMoa1800820 29897841

[B34] Tonto P. B. Gallon S. Alatrash R. Ding Y. Wang W.-K. Herrera B. B. (2025). Pre-existing YFV-17D immunity mediates T cell cross-protection against dengue virus serotype 2 infection in mice. Commun. Biol. 8, 1334. 10.1038/s42003-025-08793-3 40897800 PMC12405563

[B35] Tricou V. Sáez-Llorens X. Yu D. Rivera L. Jimeno J. Villarreal A. C. (2020). Safety and immunogenicity of a tetravalent dengue vaccine in children aged 2–17 years: a randomised, placebo-controlled, phase 2 trial. Lancet 395, 1434–1443. 10.1016/S0140-6736(20)30556-0 32197107

[B36] Tricou V. Winkle P. J. Tharenos L. M. Rauscher M. Escudero I. Hoffman E. (2023). Consistency of immunogenicity in three consecutive lots of a tetravalent dengue vaccine candidate (TAK-003): a randomized placebo-controlled trial in US adults. Vaccine 41, 6999–7006. 10.1016/j.vaccine.2023.09.049 37884415

[B37] Tricou V. Yu D. Reynales H. Biswal S. Saez-Llorens X. Sirivichayakul C. (2024). Long-term efficacy and safety of a tetravalent dengue vaccine (TAK-003): 4·5-Year results from a phase 3, randomised, double-blind, placebo-controlled trial. Lancet Glob. Health 12, e257–e270. 10.1016/S2214-109X(23)00522-3 38245116

[B38] Villar L. Dayan G. H. Arredondo-García J. L. Rivera D. M. Cunha R. Deseda C. (2015). Efficacy of a tetravalent dengue vaccine in children in Latin America. N. Engl. J. Med. 372, 113–123. 10.1056/NEJMoa1411037 25365753

[B39] Wilder-Smith A. (2024). TAK-003 dengue vaccine as a new tool to mitigate dengue in countries with a high disease burden. Lancet Glob. Health 12, e179–e180. 10.1016/S2214-109X(23)00590-9 38245106

[B40] Wilder-Smith A. Smith P. G. Luo R. Kelly-Cirino C. Curry D. Larson H. (2019). Pre-vaccination screening strategies for the use of the CYD-TDV dengue vaccine: a meeting report. Vaccine 37, 5137–5146. 10.1016/j.vaccine.2019.07.016 31377079

[B41] Winter C. (2011). MedDRA in pharmacovigilance – industry perspective. Beijing: SFDA-ICH MedDRA Workshop.

[B42] Yang Y. Meng Y. Halloran M. E. Longini I. M. (2018). Dependency of vaccine efficacy on preexposure and age: a closer look at a tetravalent dengue vaccine. Clin. Infect. Dis. 66, 178–184. 10.1093/cid/cix766 29020332 PMC5850009

[B43] Zhang X. Feng Y. Li F. Ding J. Tahseen D. Hinojosa E. (2024). Evaluating MedDRA-to-ICD terminology mappings. BMC Med. Inf. Decis. Mak. 23, 299. 10.1186/s12911-023-02375-1 38326827 PMC10851449

