# Supplementary material for: Post-marketing safety profile of dengue vaccines CYD-TDV and TAK-003: analysis of adverse event reports from a European database
Source: Front Pharmacol. 2026 Feb 20;17:1789762. doi: 10.3389/fphar.2026.1789762 (PMC12963055; doi:10.3389/fphar.2026.1789762)
Supplement: Supplementary file 1 [file Table1.docx]

**Supplementary Material Table S1.** Disproportionalities in TAK-003-related ICSRS compared to CYD-TDV stratified by PT.

| **PTs** | **TAK-003**  **N** | **CYD-TDV**  **N** | **TAK-003 vs. CYD-TDV**  **ROR [95%CI]** |
| --- | --- | --- | --- |
| Pruritus | 138 | 9 | 4.81 [2.43;9.50] |
| Angioedema | 74 | 6 | 3.74 [1.62;8.65] |
| Urticaria | 97 | 9 | 3.30 [1.65;6.57] |
| Incorrect route of product administration | 82 | 8 | 3.11 [1.50;6.48] |
| Rash pruritic | 41 | 4 | 3.06 [1.09;8.59] |
| Tachycardia | 28 | 3 | 2.77 [0.84;9.16] |
| Paraesthesia | 37 | 4 | 2.76 [0.98;7.77] |
| Fatigue | 204 | 26 | 2.48 [1.63;3.77] |
| Erythema | 56 | 7 | 2.40 [1.09;5.29] |
| Vaccination site pain | 44 | 6 | 2.19 [0.93;5.16] |
| Myalgia | 243 | 43 | 1.77 [1.26;2.48] |
| Arthralgia | 190 | 36 | 1.62 [1.12;2.34] |
| Hyperhidrosis | 21 | 4 | 1.55 [0.53;4.54] |
| Chills | 95 | 19 | 1.50 [0.91;2.48] |
| Syncope | 97 | 20 | 1.45 [0.89;2.37] |
| Malaise | 154 | 36 | 1.28 [0.88;1.87] |
| Rash maculo-papular | 13 | 3 | 1.28 [0.36;4.50] |
| Rash | 258 | 65 | 1.20 [0.89;1.60] |
| Ocular hyperaemia | 22 | 6 | 1.08 [0.44;2.68] |
| Cyanosis | 11 | 3 | 1.08 [0.30;3.88] |
| Joint swelling | 18 | 5 | 1.06 [0.39;2.87] |
| Rash papular | 10 | 3 | 0.98 [0.27;3.58] |
| Vertigo | 10 | 3 | 0.98 [0.27;3.58] |
| Nausea | 118 | 41 | 0.84 [0.58;1.21] |
| Inappropriate schedule of product administration | 14 | 5 | 0.82 [0.29;2.29] |
| Influenza | 8 | 3 | 0.78 [0.21;2.96] |
| Leukopenia | 13 | 5 | 0.76 [0.27;2.15] |
| Peripheral swelling | 15 | 6 | 0.73 [0.28;1.90] |
| Face oedema | 27 | 11 | 0.72 [0.35;1.46] |
| Sneezing | 17 | 7 | 0.71 [0.29;1.73] |
| Lymphadenopathy | 34 | 14 | 0.71 [0.38;1.33] |
| Rash erythematous | 12 | 5 | 0.70 [0.25;2.01] |
| Encephalitis | 7 | 3 | 0.69 [0.18;2.66] |
| Eye pain | 55 | 24 | 0.66 [0.41;1.08] |
| Abdominal discomfort | 9 | 4 | 0.66 [0.20;2.15] |
| Dysphagia | 11 | 5 | 0.64 [0.22;1.86] |
| Pain in extremity | 48 | 22 | 0.63 [0.38;1.06] |
| Petechiae | 21 | 10 | 0.61 [0.29;1.31] |
| Flushing | 6 | 3 | 0.59 [0.15;2.35] |
| Feeling cold | 8 | 4 | 0.59 [0.18;1.96] |
| Hypotonia | 8 | 4 | 0.59 [0.18;1.96] |
| Somnolence | 39 | 21 | 0.54 [0.31;0.92] |
| Thrombocytopenia | 25 | 14 | 0.52 [0.27;1.00] |
| Hypersensitivity | 32 | 18 | 0.51 [0.29;0.92] |
| Product administered to patient of inappropriate age | 7 | 4 | 0.51 [0.15;1.76] |
| Hyperpyrexia | 5 | 3 | 0.49 [0.12;2.05] |
| Toothache | 5 | 3 | 0.49 [0.12;2.05] |
| Rhinorrhoea | 15 | 9 | 0.49 [0.21;1.12] |
| Bone pain | 8 | 5 | 0.47 [0.15;1.44] |
| Throat tightness | 9 | 6 | 0.44 [0.16;1.24] |
| Headache | 354 | 190 | 0.43 [0.35;0.54] |
| Tremor | 11 | 8 | 0.40 [0.16;1.00] |
| Pain | 66 | 46 | 0.40 [0.27;0.59] |
| Circulatory collapse | 4 | 3 | 0.39 [0.09;1.75] |
| Palpitations | 6 | 5 | 0.35 [0.11;1.15] |
| Oropharyngeal pain | 32 | 26 | 0.35 [0.21;0.59] |
| Hypotension | 20 | 17 | 0.34 [0.18;0.65] |
| Pallor | 29 | 25 | 0.33 [0.19;0.57] |
| Cough | 59 | 51 | 0.32 [0.22;0.47] |
| Anaphylactic reaction | 119 | 99 | 0.31 [0.23;0.41] |
| Pyrexia | 358 | 238 | 0.30 [0.24;0.37] |
| Dizziness | 58 | 53 | 0.30 [0.20;0.44] |
| Acute respiratory distress syndrome | 3 | 3 | 0.29 [0.06;1.46] |
| Alopecia | 3 | 3 | 0.29 [0.06;1.46] |
| Cardio-respiratory arrest | 3 | 3 | 0.29 [0.06;1.46] |
| Dysgeusia | 3 | 3 | 0.29 [0.06;1.46] |
| Feeling abnormal | 3 | 3 | 0.29 [0.06;1.46] |
| Lymphadenitis | 3 | 3 | 0.29 [0.06;1.46] |
| Vaginal haemorrhage | 3 | 3 | 0.29 [0.06;1.46] |
| Agitation | 4 | 4 | 0.29 [0.07;1.17] |
| Balance disorder | 4 | 4 | 0.29 [0.07;1.17] |
| Heart rate increased | 4 | 4 | 0.29 [0.07;1.17] |
| Eye swelling | 5 | 5 | 0.29 [0.08;1.01] |
| Tonsillitis | 5 | 5 | 0.29 [0.08;1.01] |
| Swelling face | 11 | 11 | 0.29 [0.12;0.67] |
| Nasopharyngitis | 17 | 17 | 0.29 [0.15;0.57] |
| Back pain | 17 | 18 | 0.27 [0.14;0.53] |
| Insomnia | 7 | 8 | 0.25 [0.09;0.70] |
| Diarrhoea | 46 | 51 | 0.25 [0.16;0.37] |
| Fall | 5 | 6 | 0.24 [0.07;0.80] |
| Heavy menstrual bleeding | 5 | 6 | 0.24 [0.07;0.80] |
| Asthenia | 58 | 65 | 0.24 [0.16;0.34] |
| Seizure | 33 | 40 | 0.23 [0.14;0.37] |
| Abortion spontaneous | 3 | 4 | 0.22 [0.05;0.98] |
| Hepatitis | 3 | 4 | 0.22 [0.05;0.98] |
| Movement disorder | 3 | 4 | 0.22 [0.05;0.98] |
| Oxygen saturation decreased | 3 | 4 | 0.22 [0.05;0.98] |
| Chest pain | 17 | 23 | 0.21 [0.11;0.40] |
| Anxiety | 4 | 6 | 0.19 [0.05;0.69] |
| Paralysis | 4 | 6 | 0.19 [0.05;0.69] |
| Infection | 6 | 9 | 0.19 [0.07;0.55] |
| Blood pressure decreased | 8 | 12 | 0.19 [0.08;0.47] |
| Feeling hot | 9 | 14 | 0.18 [0.08;0.43] |
| Dyspnoea | 76 | 102 | 0.18 [0.13;0.25] |
| Musculoskeletal stiffness | 7 | 11 | 0.18 [0.07;0.48] |
| Visual impairment | 5 | 8 | 0.18 [0.06;0.56] |
| Lethargy | 8 | 13 | 0.18 [0.07;0.43] |
| Skin discolouration | 6 | 10 | 0.17 [0.06;0.48] |
| Exposure during pregnancy | 7 | 12 | 0.17 [0.07;0.43] |
| Oedema | 7 | 12 | 0.17 [0.07;0.43] |
| Neck pain | 6 | 11 | 0.16 [0.06;0.43] |
| Vision blurred | 7 | 13 | 0.16 [0.06;0.39] |
| Contusion | 4 | 8 | 0.15 [0.04;0.48] |
| Oedema peripheral | 9 | 18 | 0.14 [0.06;0.32] |
| Hypoaesthesia | 9 | 19 | 0.13 [0.06;0.30] |
| Vomiting | 62 | 113 | 0.13 [0.09;0.18] |
| Dehydration | 4 | 10 | 0.12 [0.04;0.37] |
| Chest discomfort | 7 | 18 | 0.11 [0.05;0.27] |
| Abdominal pain | 41 | 97 | 0.10 [0.07;0.15] |
| Abdominal pain upper | 16 | 43 | 0.10 [0.06;0.18] |
| Dengue haemorrhagic fever | 8 | 23 | 0.10 [0.04;0.22] |
| Irritability | 5 | 15 | 0.10 [0.03;0.26] |
| Gait disturbance | 8 | 24 | 0.09 [0.04;0.21] |
| Epistaxis | 9 | 28 | 0.09 [0.04;0.19] |
| Decreased appetite | 17 | 51 | 0.09 [0.05;0.16] |
| Sepsis | 4 | 13 | 0.09 [0.03;0.27] |
| Gastroenteritis | 3 | 10 | 0.09 [0.02;0.32] |
| Restlessness | 3 | 10 | 0.09 [0.02;0.32] |
| Vaccination failure | 13 | 42 | 0.08 [0.04;0.16] |
| Abdominal distension | 4 | 14 | 0.08 [0.03;0.25] |
| Loss of consciousness | 11 | 39 | 0.08 [0.04;0.15] |
| Swelling | 6 | 27 | 0.06 [0.03;0.15] |
| Haematemesis | 4 | 19 | 0.06 [0.02;0.18] |
| Platelet count decreased | 5 | 24 | 0.06 [0.02;0.15] |
| Pneumonia | 9 | 45 | 0.05 [0.03;0.11] |
| Dengue fever | 59 | 222 | 0.05 [0.03;0.06] |
| Illness | 4 | 53 | 0.02 [0.01;0.06] |
| Injection site erythema | 78 | 1 |  |
| Injection site pain | 71 |  |  |
| Injection site inflammation | 65 |  |  |
| Injection site warmth | 55 |  |  |
| Rash macular | 40 | 2 |  |
| Injection site swelling | 35 |  |  |
| Influenza like illness | 35 | 2 |  |
| Injection site pruritus | 33 |  |  |
| Limb discomfort | 33 | 2 |  |
| Injection site haematoma | 30 |  |  |
| Eyelid oedema | 26 | 1 |  |
| Vaccination site erythema | 23 | 2 |  |
| Anaphylactic shock | 20 | 1 |  |
| Body temperature increased | 17 |  |  |
| Breakthrough dengue fever | 16 |  |  |
| Vaccine induced antibody absent | 15 |  |  |
| Throat irritation | 13 | 2 |  |
| Facial paralysis | 12 |  |  |
| Vaccination site pruritus | 12 | 1 |  |
| Dermatitis allergic | 11 | 1 |  |
| Vaccination site swelling | 11 |  |  |
| Laryngeal oedema | 10 |  |  |
| Nasal congestion | 10 | 1 |  |
| Vaccination site reaction | 10 | 1 |  |
| Eye oedema | 9 |  |  |
| General physical health deterioration | 9 | 2 |  |
| Periorbital oedema | 9 | 1 |  |
| Product dose omission issue | 9 | 1 |  |
| Vaccination site rash | 9 | 2 |  |
| Erythema multiforme | 8 |  |  |
| Lack of vaccination site rotation | 8 |  |  |
| Dysphonia | 8 | 2 |  |
| Papule | 8 |  |  |
| Eye pruritus | 8 | 1 |  |
| Viraemia | 8 |  |  |
| Wheezing | 8 |  |  |
| Immune thrombocytopenia | 8 | 1 |  |
| Muscle spasms | 8 | 2 |  |
| Burning sensation | 7 |  |  |
| Guillain-Barre syndrome | 7 |  |  |
| Contraindicated product administered | 7 | 1 |  |
| Skin reaction | 7 |  |  |
| Hyperaemia | 7 | 1 |  |
| Muscular weakness | 7 | 2 |  |
| Cold sweat | 6 |  |  |
| Hypothermia | 6 |  |  |
| Migraine | 6 |  |  |
| Myocarditis | 6 |  |  |
| Oropharyngeal discomfort | 6 |  |  |
| Polyarthritis | 6 |  |  |
| Skin plaque | 6 |  |  |
| Tension headache | 6 |  |  |
| Vaccine viraemia | 6 |  |  |
| Monoplegia | 6 | 1 |  |
| Myositis | 6 | 1 |  |
| Vaccination site warmth | 6 | 1 |  |
| C-reactive protein increased | 5 |  |  |
| Arthritis | 5 | 1 |  |
| Blister | 5 | 2 |  |
| Haematoma | 5 |  |  |
| Injection site urticaria | 5 |  |  |
| Lip oedema | 5 |  |  |
| Neuralgia | 5 |  |  |
| Night sweats | 5 |  |  |
| Presyncope | 5 |  |  |
| Respiratory distress | 5 |  |  |
| Vaccination site oedema | 5 |  |  |
| Vaccination site urticaria | 5 |  |  |
| Immunisation reaction | 5 | 2 |  |
| Odynophagia | 5 | 2 |  |
| Bradycardia | 4 |  |  |
| Abdominal pain lower | 4 | 1 |  |
| Brain fog | 4 |  |  |
| Discouragement | 4 |  |  |
| Arrhythmia | 4 | 1 |  |
| Disturbance in attention | 4 |  |  |
| Drug ineffective | 4 |  |  |
| Dyspnoea at rest | 4 |  |  |
| Ear discomfort | 4 |  |  |
| Blood pressure increased | 4 | 2 |  |
| Eczema | 4 |  |  |
| Injection site discolouration | 4 |  |  |
| Confusional state | 4 | 2 |  |
| Oral pruritus | 4 |  |  |
| Peripheral coldness | 4 |  |  |
| Tachypnoea | 4 |  |  |
| Type III immune complex mediated reaction | 4 |  |  |
| Herpes zoster | 4 | 1 |  |
| Joint stiffness | 4 | 2 |  |
| Photophobia | 4 | 1 |  |
| Tinnitus | 4 | 1 |  |
| White blood cell count decreased | 4 | 1 |  |
| Administration site bruise | 3 |  |  |
| Bronchitis | 3 |  |  |
| Cognitive disorder | 3 |  |  |
| Depressed level of consciousness | 3 |  |  |
| Apathy | 3 | 1 |  |
| Dyspnoea exertional | 3 |  |  |
| Ear pain | 3 |  |  |
| Facial pain | 3 |  |  |
| Cardiac failure | 3 | 1 |  |
| Head discomfort | 3 |  |  |
| Injection site induration | 3 |  |  |
| Dermatitis bullous | 3 | 2 |  |
| Oral mucosal blistering | 3 |  |  |
| Pain of skin | 3 |  |  |
| Pancreatitis acute | 3 |  |  |
| Paraesthesia oral | 3 |  |  |
| Product availability issue | 3 |  |  |
| Pulmonary embolism | 3 |  |  |
| Sensitive skin | 3 |  |  |
| Serum sickness | 3 |  |  |
| Severe acute respiratory syndrome | 3 |  |  |
| Vaccination error | 3 |  |  |
| Vaccination site bruising | 3 |  |  |
| Vaccine interaction | 3 |  |  |
| Musculoskeletal chest pain | 3 | 1 |  |
| Periorbital swelling | 3 | 1 |  |
| Pharyngitis | 3 | 1 |  |
| Pustule | 3 | 2 |  |
| Transaminases increased | 3 | 1 |  |
| Vaccination site discolouration | 3 | 1 |  |
| Vaccination site haematoma | 3 | 1 |  |
| Albumin globulin ratio abnormal | 2 |  |  |
| Allergy to vaccine | 2 | 2 |  |
| Aphasia | 2 | 5 |  |
| Benign prostatic hyperplasia | 2 |  |  |
| Blindness | 2 | 10 |  |
| Body temperature abnormal | 2 |  |  |
| Bronchospasm | 2 |  |  |
| Cardio-respiratory distress | 2 |  |  |
| Carpal tunnel syndrome | 2 |  |  |
| Condition aggravated | 2 | 93 |  |
| Conjunctival oedema | 2 |  |  |
| Deafness neurosensory | 2 |  |  |
| Deafness unilateral | 2 |  |  |
| Dengue virus test positive | 2 | 51 |  |
| Depressed mood | 2 | 2 |  |
| Discomfort | 2 | 1 |  |
| Drug eruption | 2 |  |  |
| Dysentery | 2 |  |  |
| Dysstasia | 2 | 14 |  |
| Ear swelling | 2 | 2 |  |
| Extra dose administered | 2 |  |  |
| Food aversion | 2 |  |  |
| Gastrooesophageal reflux disease | 2 | 2 |  |
| Haematochezia | 2 | 7 |  |
| Haematuria | 2 | 1 |  |
| Hallucination | 2 | 3 |  |
| Herpes virus infection | 2 |  |  |
| Hordeolum | 2 |  |  |
| Hypoaesthesia oral | 2 |  |  |
| Intentional dose omission | 2 |  |  |
| Lip swelling | 2 | 1 |  |
| Liver function test increased | 2 |  |  |
| Local reaction | 2 |  |  |
| Locomotive syndrome | 2 |  |  |
| Lymphocytosis | 2 |  |  |
| Lymphoedema | 2 |  |  |
| Menstruation irregular | 2 |  |  |
| Muscle fatigue | 2 | 1 |  |
| Musculoskeletal discomfort | 2 |  |  |
| Nasal obstruction | 2 |  |  |
| Nephrolithiasis | 2 | 1 |  |
| Neuritis | 2 |  |  |
| Neurological symptom | 2 |  |  |
| Nightmare | 2 |  |  |
| Ocular discomfort | 2 |  |  |
| Oedema mouth | 2 |  |  |
| Orthostatic hypotension | 2 | 1 |  |
| Peripheral vascular disorder | 2 |  |  |
| Pharyngeal oedema | 2 |  |  |
| Pharyngeal swelling | 2 | 1 |  |
| Photosensitivity reaction | 2 |  |  |
| Pleural effusion | 2 | 6 |  |
| Premature labour | 2 |  |  |
| Pruritus allergic | 2 |  |  |
| Pulmonary oedema | 2 | 11 |  |
| Pulse abnormal | 2 | 2 |  |
| Purpura | 2 | 3 |  |
| Pyelonephritis | 2 |  |  |
| Rash morbilliform | 2 |  |  |
| Rash vesicular | 2 | 1 |  |
| Raynaud's phenomenon | 2 |  |  |
| Respiratory disorder | 2 |  |  |
| Respiratory symptom | 2 |  |  |
| Rhabdomyolysis | 2 |  |  |
| Salivary hypersecretion | 2 | 5 |  |
| Shock | 2 | 3 |  |
| Sinus pain | 2 |  |  |
| Sinusitis | 2 | 1 |  |
| Skin irritation | 2 |  |  |
| Skin warm | 2 |  |  |
| Stevens-Johnson syndrome | 2 |  |  |
| Stomatitis | 2 |  |  |
| Throat clearing | 2 |  |  |
| Thrombosis | 2 | 4 |  |
| Tongue blistering | 2 |  |  |
| Type I hypersensitivity | 2 |  |  |
| Unresponsive to stimuli | 2 | 3 |  |
| Urinary retention | 2 |  |  |
| Urticaria chronic | 2 |  |  |
| Urticaria papular | 2 |  |  |
| Vaccination site abscess | 2 |  |  |
| Vaccination site hypersensitivity | 2 |  |  |
| Vaccination site lymphadenopathy | 2 |  |  |
| Vasculitis | 2 |  |  |
| Ventricular septal defect | 2 |  |  |
| Viral infection | 2 | 4 |  |
| Viral rash | 2 | 1 |  |
| Abnormal behaviour | 1 | 9 |  |
| Abnormal faeces | 1 |  |  |
| Abortion | 1 | 1 |  |
| Abscess | 1 | 3 |  |
| Abulia | 1 |  |  |
| Acne | 1 | 2 |  |
| Acute abdomen | 1 |  |  |
| Acute kidney injury | 1 | 2 |  |
| Acute myocardial infarction | 1 | 1 |  |
| Adenoviral conjunctivitis | 1 |  |  |
| Administration site indentation | 1 |  |  |
| Administration site oedema | 1 |  |  |
| Allodynia | 1 |  |  |
| Anaesthesia oral | 1 |  |  |
| Angina pectoris | 1 |  |  |
| Angiodermatitis | 1 |  |  |
| Angiopathy | 1 |  |  |
| Aortic disorder | 1 |  |  |
| Aphthous ulcer | 1 |  |  |
| Application site pain | 1 | 1 |  |
| Application site pruritus | 1 |  |  |
| Arthritis reactive | 1 |  |  |
| Arthropathy | 1 | 1 |  |
| Ascites | 1 | 3 |  |
| Asthma | 1 | 3 |  |
| Ataxia | 1 |  |  |
| Atrial septal defect | 1 |  |  |
| Atrioventricular block first degree | 1 |  |  |
| Autoimmune thyroiditis | 1 |  |  |
| Autonomic nervous system imbalance | 1 |  |  |
| Balanoposthitis | 1 |  |  |
| Band sensation | 1 |  |  |
| Bartholin's cyst | 1 |  |  |
| Bell's palsy | 1 |  |  |
| Blindness day | 1 |  |  |
| Blindness transient | 1 |  |  |
| Blindness unilateral | 1 |  |  |
| Blood creatine phosphokinase increased | 1 |  |  |
| Blood lactic acid increased | 1 |  |  |
| Blood pressure abnormal | 1 |  |  |
| Body temperature | 1 |  |  |
| Bone neoplasm | 1 |  |  |
| Borrelia test positive | 1 |  |  |
| Breast pain | 1 |  |  |
| Bursitis | 1 |  |  |
| Cardiac myxoma | 1 |  |  |
| Cardiogenic shock | 1 | 2 |  |
| Cardiomegaly | 1 | 9 |  |
| Cataract | 1 |  |  |
| Catarrh | 1 |  |  |
| Cerebellar syndrome | 1 |  |  |
| Cerebrovascular accident | 1 | 6 |  |
| Cervix dystocia | 1 |  |  |
| Choking sensation | 1 |  |  |
| Chromaturia | 1 | 1 |  |
| Circumstance or information capable of leading to medication error | 1 |  |  |
| Coagulopathy | 1 |  |  |
| Complex regional pain syndrome | 1 |  |  |
| Concussion | 1 |  |  |
| Conjunctival hyperaemia | 1 |  |  |
| Conjunctivitis allergic | 1 |  |  |
| Constipation | 1 | 1 |  |
| Coordination abnormal | 1 |  |  |
| Costochondritis | 1 |  |  |
| COVID-19 | 1 | 1 |  |
| Craniocerebral injury | 1 |  |  |
| Craniofacial fracture | 1 |  |  |
| Cutaneous symptom | 1 |  |  |
| Cutaneous vasculitis | 1 |  |  |
| Cystitis haemorrhagic | 1 |  |  |
| Cytokine storm | 1 |  |  |
| Dactylitis | 1 |  |  |
| Deafness | 1 | 1 |  |
| Death | 1 | 89 |  |
| Decreased immune responsiveness | 1 |  |  |
| Deep vein thrombosis | 1 |  |  |
| Delirium | 1 | 10 |  |
| Demyelination | 1 |  |  |
| Dengue shock syndrome | 1 | 1 |  |
| Derealisation | 1 |  |  |
| Dermatitis | 1 |  |  |
| Dermatitis acneiform | 1 |  |  |
| Diabetes mellitus | 1 | 3 |  |
| Diabetic ketoacidosis | 1 | 3 |  |
| Diarrhoea haemorrhagic | 1 | 3 |  |
| Diplacusis | 1 |  |  |
| Diplegia | 1 |  |  |
| Diplopia | 1 |  |  |
| Disease recurrence | 1 |  |  |
| Disorientation | 1 |  |  |
| Dissociation | 1 |  |  |
| Diverticulum intestinal | 1 |  |  |
| Drooling | 1 |  |  |
| Dry mouth | 1 | 1 |  |
| Dysarthria | 1 | 5 |  |
| Dysbiosis | 1 |  |  |
| Dysmenorrhoea | 1 | 1 |  |
| Dysphemia | 1 |  |  |
| Dyspraxia | 1 |  |  |
| Ecchymosis | 1 | 1 |  |
| Electrolyte imbalance | 1 | 1 |  |
| Encephalitis bacterial | 1 |  |  |
| Encephalopathy | 1 | 2 |  |
| Epicondylitis | 1 |  |  |
| Epilepsy | 1 |  |  |
| Eructation | 1 |  |  |
| Erythema nodosum | 1 |  |  |
| Extensive swelling of vaccinated limb | 1 |  |  |
| Eye discharge | 1 | 1 |  |
| Eye disorder | 1 | 2 |  |
| Eye irritation | 1 |  |  |
| Eyelid haematoma | 1 |  |  |
| Eyelid pain | 1 |  |  |
| Eyelid ptosis | 1 |  |  |
| Facial paresis | 1 |  |  |
| Faeces discoloured | 1 | 4 |  |
| Febrile convulsion | 1 | 1 |  |
| Feeling drunk | 1 |  |  |
| Flatulence | 1 | 1 |  |
| Focal dyscognitive seizures | 1 |  |  |
| Foetal anaemia | 1 |  |  |
| Foetal cardiac disorder | 1 |  |  |
| Foetal cystic hygroma | 1 |  |  |
| Foetal death | 1 | 1 |  |
| Foetal exposure during pregnancy | 1 | 1 |  |
| Foetal growth restriction | 1 |  |  |
| Folliculitis | 1 |  |  |
| Formication | 1 |  |  |
| Fracture | 1 |  |  |
| Gait inability | 1 | 6 |  |
| Gastritis | 1 | 3 |  |
| Gastrointestinal pain | 1 |  |  |
| Generalised oedema | 1 | 4 |  |
| Genital lesion | 1 |  |  |
| Gingival blister | 1 |  |  |
| Gingival discolouration | 1 |  |  |
| Gingival injury | 1 |  |  |
| Gingival pain | 1 | 1 |  |
| Glaucoma | 1 |  |  |
| Glomerulonephritis minimal lesion | 1 |  |  |
| Haemangioma | 1 |  |  |
| Haemoglobin decreased | 1 | 4 |  |
| Haemoptysis | 1 | 10 |  |
| Haemorrhage in pregnancy | 1 |  |  |
| Haemorrhage subcutaneous | 1 |  |  |
| Haemorrhagic necrotic pancreatitis | 1 |  |  |
| Haemorrhoids thrombosed | 1 |  |  |
| Head injury | 1 | 1 |  |
| Heart rate decreased | 1 | 4 |  |
| Hemiparaesthesia | 1 |  |  |
| Henoch-Schonlein purpura | 1 | 1 |  |
| Hepatic cytolysis | 1 |  |  |
| Hepatomegaly | 1 | 6 |  |
| Herpes simplex reactivation | 1 |  |  |
| Hiccups | 1 |  |  |
| Hodgkin's disease | 1 |  |  |
| Hydrops foetalis | 1 |  |  |
| Hyperaesthesia eye | 1 |  |  |
| Hyperbilirubinaemia | 1 |  |  |
| Hypersensitivity pneumonitis | 1 |  |  |
| Hypersomnia | 1 | 3 |  |
| Hypersplenism | 1 |  |  |
| Hypertension | 1 | 6 |  |
| Hypertensive crisis | 1 |  |  |
| Hypertransaminasaemia | 1 |  |  |
| Hypoacusis | 1 | 1 |  |
| Hypokinesia | 1 | 5 |  |
| Hypophagia | 1 |  |  |
| Hyporeflexia | 1 |  |  |
| Hypothyroidism | 1 | 1 |  |
| Hypotonic-hyporesponsive episode | 1 |  |  |
| Hypovolaemic shock | 1 | 7 |  |
| Hypoxic-ischaemic encephalopathy | 1 |  |  |
| Impaired quality of life | 1 |  |  |
| Impatience | 1 | 1 |  |
| Incomplete course of vaccination | 1 | 1 |  |
| Incontinence | 1 | 1 |  |
| Incorrect dose administered | 1 |  |  |
| Increased appetite | 1 |  |  |
| Increased upper airway secretion | 1 |  |  |
| Infarction | 1 |  |  |
| Inflammation | 1 | 23 |  |
| Initial insomnia | 1 |  |  |
| Injection site oedema | 1 |  |  |
| Injection site paraesthesia | 1 |  |  |
| Injection site rash | 1 |  |  |
| Injection site vesicles | 1 |  |  |
| Intermenstrual bleeding | 1 |  |  |
| Interstitial lung disease | 1 | 1 |  |
| Intervertebral disc protrusion | 1 |  |  |
| Iron deficiency | 1 |  |  |
| Ischaemic stroke | 1 |  |  |
| Jaundice | 1 | 4 |  |
| Joint effusion | 1 |  |  |
| Klebsiella infection | 1 |  |  |
| Left ventricular dysfunction | 1 |  |  |
| Leprosy | 1 |  |  |
| Leukocytosis | 1 | 2 |  |
| Leukoencephalopathy | 1 |  |  |
| Lip discolouration | 1 | 5 |  |
| Lip pruritus | 1 | 1 |  |
| Listless | 1 |  |  |
| Liver disorder | 1 |  |  |
| Liver function test abnormal | 1 |  |  |
| Localised oedema | 1 |  |  |
| Loss of personal independence in daily activities | 1 | 1 |  |
| Lung infiltration | 1 |  |  |
| Lymph node palpable | 1 |  |  |
| Lymphoma | 1 | 1 |  |
| Lymphopenia | 1 | 1 |  |
| Macular oedema | 1 |  |  |
| Mass | 1 | 6 |  |
| Maternal exposure before pregnancy | 1 |  |  |
| Mechanical urticaria | 1 |  |  |
| Memory impairment | 1 | 2 |  |
| Meningitis meningococcal | 1 |  |  |
| Mental impairment | 1 |  |  |
| Mesenteric vein thrombosis | 1 |  |  |
| Middle insomnia | 1 |  |  |
| Mite allergy | 1 |  |  |
| Mitral valve disease | 1 |  |  |
| Mood altered | 1 |  |  |
| Mouth swelling | 1 |  |  |
| Mouth ulceration | 1 |  |  |
| Mucosa vesicle | 1 |  |  |
| Multiple allergies | 1 |  |  |
| Multiple sclerosis | 1 |  |  |
| Muscle contractions involuntary | 1 |  |  |
| Muscle swelling | 1 |  |  |
| Muscle tightness | 1 |  |  |
| Muscle twitching | 1 | 1 |  |
| Musculoskeletal pain | 1 |  |  |
| Mycoplasma infection | 1 |  |  |
| Myelin oligodendrocyte glycoprotein antibody-associated disease | 1 |  |  |
| Myelitis | 1 |  |  |
| Myoglobin blood increased | 1 |  |  |
| Myopericarditis | 1 |  |  |
| Nasal oedema | 1 | 1 |  |
| Nervousness | 1 |  |  |
| Neuralgic amyotrophy | 1 |  |  |
| Neutropenia | 1 |  |  |
| Neutrophil count decreased | 1 | 1 |  |
| No adverse event | 1 |  |  |
| Nodule | 1 |  |  |
| Ocular myasthenia | 1 |  |  |
| Oesophageal discomfort | 1 |  |  |
| Off label use | 1 | 4 |  |
| Oligohydramnios | 1 |  |  |
| Ophthalmic herpes zoster | 1 |  |  |
| Optic neuritis | 1 |  |  |
| Oropharyngeal blistering | 1 |  |  |
| Oropharyngeal erythema | 1 | 2 |  |
| Oropouche fever | 1 |  |  |
| Otitis media acute | 1 |  |  |
| Pain in jaw | 1 | 2 |  |
| Painful respiration | 1 |  |  |
| Palatal oedema | 1 |  |  |
| Paresis | 1 | 1 |  |
| Parotitis | 1 |  |  |
| Partial seizures | 1 |  |  |
| Parvovirus infection | 1 |  |  |
| Pelvic fluid collection | 1 |  |  |
| Pericardial effusion | 1 |  |  |
| Perioral dermatitis | 1 |  |  |
| Periorbital pain | 1 |  |  |
| Pharyngeal inflammation | 1 |  |  |
| Pigmentation disorder | 1 | 2 |  |
| Plasma cell myeloma | 1 |  |  |
| Platelet count increased | 1 | 2 |  |
| Pneumonia bacterial | 1 |  |  |
| Pneumonia mycoplasmal | 1 |  |  |
| Pneumonia necrotising | 1 |  |  |
| Poor quality sleep | 1 |  |  |
| Poor venous access | 1 |  |  |
| Portal vein thrombosis | 1 |  |  |
| Post herpetic neuralgia | 1 |  |  |
| Prerenal failure | 1 |  |  |
| Prescription drug used without a prescription | 1 |  |  |
| Product administered at inappropriate site | 1 |  |  |
| Product preparation error | 1 |  |  |
| Productive cough | 1 | 9 |  |
| Prostatitis | 1 |  |  |
| Proteinuria | 1 |  |  |
| Pruritus genital | 1 |  |  |
| Psychomotor hyperactivity | 1 |  |  |
| Psychotic disorder | 1 |  |  |
| Pubic pain | 1 |  |  |
| Pulmonary arterial hypertension | 1 |  |  |
| Pulmonary hypertension | 1 |  |  |
| Pyelitis | 1 |  |  |
| Rash rubelliform | 1 |  |  |
| Reaction to excipient | 1 |  |  |
| Renal failure | 1 | 1 |  |
| Respiratory failure | 1 | 3 |  |
| Respiratory rate increased | 1 | 1 |  |
| Respiratory tract infection | 1 | 1 |  |
| Rest regimen | 1 |  |  |
| Retinal artery occlusion | 1 |  |  |
| Retroplacental haematoma | 1 |  |  |
| Rheumatic disorder | 1 |  |  |
| Rhinitis | 1 | 1 |  |
| Routine immunisation schedule incomplete | 1 |  |  |
| Seasonal allergy | 1 |  |  |
| Secretion discharge | 1 |  |  |
| Sedation | 1 |  |  |
| Self-injurious ideation | 1 |  |  |
| Sensory disturbance | 1 |  |  |
| Sensory processing disorder | 1 |  |  |
| Shock haemorrhagic | 1 |  |  |
| Single umbilical artery | 1 |  |  |
| Sinus bradycardia | 1 | 1 |  |
| Sinus tachycardia | 1 | 1 |  |
| Skin abrasion | 1 |  |  |
| Skin exfoliation | 1 |  |  |
| Skin haemorrhage | 1 | 1 |  |
| Skin lesion | 1 |  |  |
| Skin mass | 1 |  |  |
| Skin oedema | 1 |  |  |
| Skin papilloma | 1 |  |  |
| Skin tightness | 1 |  |  |
| Sleep disorder | 1 | 2 |  |
| Slow response to stimuli | 1 |  |  |
| Speech disorder | 1 | 5 |  |
| Spinal pain | 1 | 1 |  |
| Splenomegaly | 1 | 2 |  |
| Still's disease | 1 |  |  |
| Streptococcus test positive | 1 |  |  |
| Subarachnoid haemorrhage | 1 | 1 |  |
| Sudden hearing loss | 1 |  |  |
| Suicidal ideation | 1 |  |  |
| Surgery | 1 |  |  |
| Synovial cyst | 1 |  |  |
| Synovitis | 1 |  |  |
| Tenderness | 1 |  |  |
| Tendonitis | 1 |  |  |
| Tenosynovitis | 1 |  |  |
| Therapeutic product effect prolonged | 1 |  |  |
| Thrombophlebitis | 1 |  |  |
| Thyroid dermatopathy | 1 |  |  |
| Thyroiditis acute | 1 |  |  |
| Tongue biting | 1 |  |  |
| Tongue discomfort | 1 |  |  |
| Tongue disorder | 1 | 1 |  |
| Tongue oedema | 1 |  |  |
| Tongue pruritus | 1 |  |  |
| Tongue ulceration | 1 |  |  |
| Tonsillar hypertrophy | 1 | 1 |  |
| Tracheitis | 1 |  |  |
| Transient ischaemic attack | 1 |  |  |
| Tuberculosis | 1 | 8 |  |
| Type 1 diabetes mellitus | 1 |  |  |
| Type 2 diabetes mellitus | 1 |  |  |
| Ulnar nerve palsy | 1 |  |  |
| Upper respiratory tract infection | 1 | 2 |  |
| Urinary tract candidiasis | 1 |  |  |
| Urinary tract infection | 1 | 31 |  |
| Urticarial vasculitis | 1 |  |  |
| Uterine haemorrhage | 1 |  |  |
| Vaccination site discharge | 1 |  |  |
| Vaccination site discomfort | 1 |  |  |
| Vaccination site haemorrhage | 1 |  |  |
| Vaccination site hypoaesthesia | 1 |  |  |
| Vaccination site induration | 1 | 1 |  |
| Vaccination site movement impairment | 1 |  |  |
| Vaccination site vesicles | 1 |  |  |
| Vaccine breakthrough infection | 1 |  |  |
| Vaginal ulceration | 1 |  |  |
| Varicella zoster virus infection | 1 |  |  |
| Venous thrombosis | 1 |  |  |
| Vestibular neuronitis | 1 |  |  |
| Viral myositis | 1 |  |  |
| Viral upper respiratory tract infection | 1 |  |  |
| Vomiting projectile | 1 |  |  |
| Vulva cyst | 1 |  |  |
| Vulval ulceration | 1 |  |  |
| Weight bearing difficulty | 1 |  |  |
| Weight decreased | 1 | 24 |  |
| Wound | 1 | 3 |  |
| Yellow fever | 1 |  |  |
| Erythema of eyelid |  | 1 |  |
| Abdominal abscess |  | 1 |  |
| Ear haemorrhage |  | 2 |  |
| Ear infection |  | 1 |  |
| Eating disorder |  | 1 |  |
| Encephalitis viral |  | 2 |  |
| End stage renal disease |  | 2 |  |
| Endotracheal intubation |  | 3 |  |
| Enteritis |  | 1 |  |
| Enterovirus infection |  | 1 |  |
| Dyspepsia |  | 1 |  |
| Epiglottic oedema |  | 1 |  |
| Dyskinesia |  | 4 |  |
| Exophthalmos |  | 3 |  |
| Eye colour change |  | 1 |  |
| Eye haemorrhage |  | 2 |  |
| Eye inflammation |  | 1 |  |
| Eye movement disorder |  | 7 |  |
| Feeding disorder |  | 7 |  |
| Flank pain |  | 1 |  |
| Foaming at mouth |  | 2 |  |
| Full blood count abnormal |  | 1 |  |
| Full blood count decreased |  | 1 |  |
| Eosinophil count increased |  | 1 |  |
| Diarrhoea infectious |  | 1 |  |
| Cystitis |  | 1 |  |
| Dark circles under eyes |  | 1 |  |
| Daydreaming |  | 3 |  |
| Decreased interest |  | 1 |  |
| Decubitus ulcer |  | 2 |  |
| Defaecation disorder |  | 1 |  |
| Delusion |  | 1 |  |
| Dental caries |  | 1 |  |
| Depression |  | 1 |  |
| Dysuria |  | 6 |  |
| Diabetes insipidus |  | 1 |  |
| Gallbladder enlargement |  | 1 |  |
| Diphtheria |  | 1 |  |
| Discharge |  | 1 |  |
| Discoloured vomit |  | 3 |  |
| Disease progression |  | 2 |  |
| Disseminated intravascular coagulation |  | 5 |  |
| Drug hypersensitivity |  | 1 |  |
| Drug-induced liver injury |  | 1 |  |
| Dry eye |  | 2 |  |
| Dry skin |  | 1 |  |
| Duodenal ulcer |  | 1 |  |
| Desmoplastic small round cell tumour |  | 1 |  |
| Hepatic neoplasm |  | 1 |  |
| Haemothorax |  | 1 |  |
| Hand fracture |  | 1 |  |
| Head banging |  | 1 |  |
| Heart disease congenital |  | 2 |  |
| Heart rate abnormal |  | 2 |  |
| Heart rate irregular |  | 1 |  |
| Heart sounds abnormal |  | 1 |  |
| Helicobacter infection |  | 1 |  |
| Hepatic enzyme increased |  | 1 |  |
| Fungal infection |  | 1 |  |
| Hepatic necrosis |  | 2 |  |
| Haemorrhagic arteriovenous malformation |  | 1 |  |
| Hepatic steatosis |  | 1 |  |
| Hepatitis viral |  | 1 |  |
| Hepatosplenomegaly |  | 2 |  |
| Hospitalisation |  | 3 |  |
| Hunger |  | 1 |  |
| Hydrocephalus |  | 3 |  |
| Hydronephrosis |  | 1 |  |
| Hyperchlorhydria |  | 1 |  |
| Hyperglycaemia |  | 1 |  |
| Hyperleukocytosis |  | 1 |  |
| Hepatic haemorrhage |  | 10 |  |
| Gingival bleeding |  | 13 |  |
| Crying |  | 5 |  |
| Gastric haemorrhage |  | 8 |  |
| Gastric ulcer |  | 1 |  |
| Gastrointestinal disorder |  | 8 |  |
| Gastrointestinal haemorrhage |  | 6 |  |
| Gastrointestinal perforation |  | 1 |  |
| Gastrointestinal sounds abnormal |  | 1 |  |
| Gastrointestinal ulcer |  | 1 |  |
| Gaze palsy |  | 3 |  |
| Genitals enlarged |  | 1 |  |
| Haemorrhagic varicella syndrome |  | 1 |  |
| Gestational diabetes |  | 1 |  |
| Haemorrhagic diathesis |  | 1 |  |
| Gingival swelling |  | 1 |  |
| Haematocrit increased |  | 1 |  |
| Haematological infection |  | 1 |  |
| Haematology test abnormal |  | 1 |  |
| Haematopoietic neoplasm |  | 1 |  |
| Haemodialysis |  | 1 |  |
| Haemoglobin abnormal |  | 1 |  |
| Haemorrhage |  | 33 |  |
| Haemorrhage intracranial |  | 3 |  |
| Furuncle |  | 1 |  |
| Germ cell neoplasm |  | 1 |  |
| Autoimmune disorder |  | 1 |  |
| Aneurysm |  | 1 |  |
| Anger |  | 8 |  |
| Anisocoria |  | 1 |  |
| Antibody test negative |  | 4 |  |
| Aphonia |  | 2 |  |
| Aplastic anaemia |  | 2 |  |
| Appendicitis perforated |  | 3 |  |
| Arachnoid cyst |  | 1 |  |
| Arteriovenous malformation |  | 2 |  |
| Blood immunoglobulin M increased |  | 1 |  |
| Atopy |  | 1 |  |
| Anaemia |  | 8 |  |
| Azotaemia |  | 1 |  |
| Bacterial infection |  | 2 |  |
| Bacterial test positive |  | 1 |  |
| Bedridden |  | 1 |  |
| Behaviour disorder |  | 2 |  |
| Biliary colic |  | 1 |  |
| Blood creatinine increased |  | 1 |  |
| Blood disorder |  | 3 |  |
| Blood glucose decreased |  | 1 |  |
| Cyst |  | 2 |  |
| Asthmatic crisis |  | 2 |  |
| Adverse drug reaction |  | 2 |  |
| Abdominal infection |  | 1 |  |
| Abdominal mass |  | 1 |  |
| Abnormal menstrual clots |  | 1 |  |
| Abnormal organ growth |  | 2 |  |
| Abortion infected |  | 1 |  |
| Abscess drainage |  | 1 |  |
| Abscess neck |  | 1 |  |
| Acute coronary syndrome |  | 1 |  |
| Acute leukaemia |  | 3 |  |
| Acute lymphocytic leukaemia |  | 4 |  |
| Anal incontinence |  | 2 |  |
| Acute respiratory failure |  | 6 |  |
| Anal haemorrhage |  | 1 |  |
| Adverse event |  | 1 |  |
| Ageusia |  | 1 |  |
| Aggression |  | 3 |  |
| Agnosia |  | 1 |  |
| Alanine aminotransferase increased |  | 1 |  |
| Altered state of consciousness |  | 3 |  |
| Amnesia |  | 1 |  |
| Amoebiasis |  | 4 |  |
| Amputation |  | 1 |  |
| Blood potassium decreased |  | 2 |  |
| Acute myeloid leukaemia |  | 3 |  |
| Colitis |  | 2 |  |
| Cellulitis |  | 3 |  |
| Central nervous system infection |  | 1 |  |
| Cerebellar cyst |  | 1 |  |
| Cerebral disorder |  | 1 |  |
| Cerebral haemorrhage |  | 115 |  |
| Cerebral palsy |  | 1 |  |
| Cheilitis |  | 1 |  |
| Chikungunya virus infection |  | 1 |  |
| Chronic gastritis |  | 1 |  |
| Blood glucose increased |  | 1 |  |
| Coagulation time prolonged |  | 1 |  |
| Cardiac murmur |  | 2 |  |
| Colitis ischaemic |  | 1 |  |
| Coma |  | 17 |  |
| Completed suicide |  | 1 |  |
| Conjunctival haemorrhage |  | 1 |  |
| Contraindication to vaccination |  | 10 |  |
| Conversion disorder |  | 12 |  |
| Convulsions local |  | 1 |  |
| Crepitations |  | 1 |  |
| Critical illness |  | 4 |  |
| Hyperthermia |  | 1 |  |
| Cluster headache |  | 1 |  |
| Brain neoplasm |  | 3 |  |
| Blood pressure immeasurable |  | 1 |  |
| Blood urine present |  | 3 |  |
| Blood viscosity abnormal |  | 1 |  |
| Bloody discharge |  | 1 |  |
| Bone cancer |  | 1 |  |
| Bone giant cell tumour |  | 1 |  |
| Bone tuberculosis |  | 3 |  |
| Bowel movement irregularity |  | 3 |  |
| Brain abscess |  | 1 |  |
| Brain death |  | 4 |  |
| Cardiovascular disorder |  | 1 |  |
| Brain injury |  | 2 |  |
| Cardiac neoplasm unspecified |  | 1 |  |
| Brain oedema |  | 29 |  |
| Brain operation |  | 1 |  |
| Brain stem haemorrhage |  | 1 |  |
| Bronchiolitis |  | 2 |  |
| Bronchopulmonary aspergillosis |  | 1 |  |
| Cachexia |  | 1 |  |
| Cardiac arrest |  | 3 |  |
| Cardiac disorder |  | 9 |  |
| Cardiac failure congestive |  | 2 |  |
| CSF protein increased |  | 1 |  |
| Brain herniation |  | 3 |  |
| Sluggishness |  | 1 |  |
| Salivary gland enlargement |  | 1 |  |
| Scratch |  | 1 |  |
| Screaming |  | 6 |  |
| Septic encephalopathy |  | 1 |  |
| Septic shock |  | 11 |  |
| Shprintzen-Goldberg syndrome |  | 1 |  |
| Skin burning sensation |  | 1 |  |
| Skin striae |  | 1 |  |
| Skin ulcer |  | 1 |  |
| Strabismus |  | 4 |  |
| Skin wrinkling |  | 2 |  |
| Rheumatic fever |  | 1 |  |
| Smallpox |  | 2 |  |
| Snoring |  | 2 |  |
| Spinal cord neoplasm |  | 1 |  |
| Spleen congestion |  | 1 |  |
| Splenitis |  | 1 |  |
| Sputum discoloured |  | 1 |  |
| Sputum purulent |  | 1 |  |
| Staphylococcal infection |  | 1 |  |
| Staring |  | 1 |  |
| Postpartum haemorrhage |  | 1 |  |
| Skin weeping |  | 1 |  |
| Renal disorder |  | 4 |  |
| Hyperphagia |  | 1 |  |
| Procedural pain |  | 1 |  |
| Product use issue |  | 14 |  |
| Prosopagnosia |  | 1 |  |
| Prostatitis Escherichia coli |  | 1 |  |
| Pulmonary congestion |  | 4 |  |
| Pulmonary fibrosis |  | 1 |  |
| Pulmonary haemorrhage |  | 25 |  |
| Pulmonary tuberculosis |  | 1 |  |
| Pulse absent |  | 2 |  |
| Rosacea |  | 1 |  |
| Red blood cell count decreased |  | 1 |  |
| Rheumatic heart disease |  | 6 |  |
| Renal haemorrhage |  | 2 |  |
| Renal tubular dysfunction |  | 1 |  |
| Renal tubular necrosis |  | 2 |  |
| Respiration abnormal |  | 1 |  |
| Respiratory arrest |  | 4 |  |
| Respiratory rate decreased |  | 1 |  |
| Restrictive pulmonary disease |  | 1 |  |
| Resuscitation |  | 1 |  |
| Retching |  | 3 |  |
| Stridor |  | 1 |  |
| Rectal haemorrhage |  | 1 |  |
| Visual acuity reduced |  | 1 |  |
| Uterine atony |  | 1 |  |
| Vaccination complication |  | 1 |  |
| Vaccination site nodule |  | 2 |  |
| Varicella |  | 3 |  |
| Varicose vein |  | 2 |  |
| Vasodilatation |  | 2 |  |
| Vein disorder |  | 1 |  |
| Vena cava injury |  | 1 |  |
| Viral sepsis |  | 1 |  |
| Steroid dependence |  | 1 |  |
| Visceroptosis |  | 4 |  |
| Urinary incontinence |  | 3 |  |
| Vital functions abnormal |  | 1 |  |
| Vulvovaginal swelling |  | 1 |  |
| Vulvovaginal warts |  | 1 |  |
| Wheelchair user |  | 1 |  |
| White blood cell count increased |  | 2 |  |
| Wound haemorrhage |  | 1 |  |
| Wound infection |  | 1 |  |
| Yellow fever vaccine-associated neurotropic disease |  | 2 |  |
| Yellow fever vaccine-associated viscerotropic disease |  | 2 |  |
| Yellow skin |  | 4 |  |
| Visceral oedema |  | 28 |  |
| Tonsillitis bacterial |  | 1 |  |
| Stubbornness |  | 2 |  |
| Sudden death |  | 4 |  |
| Swollen tongue |  | 1 |  |
| Symptom recurrence |  | 1 |  |
| Syphilis |  | 2 |  |
| Syphilis test positive |  | 1 |  |
| Systemic lupus erythematosus |  | 4 |  |
| Systemic viral infection |  | 1 |  |
| Thirst |  | 1 |  |
| Thyroid disorder |  | 1 |  |
| Urine output decreased |  | 3 |  |
| Tonsillar exudate |  | 1 |  |
| Urine abnormality |  | 1 |  |
| Tooth abscess |  | 1 |  |
| Toxoplasmosis |  | 1 |  |
| Tracheostomy |  | 1 |  |
| Transfusion |  | 6 |  |
| Trismus |  | 2 |  |
| Tumour lysis syndrome |  | 1 |  |
| Typhoid fever |  | 6 |  |
| Unevaluable event |  | 1 |  |
| Urinary hesitation |  | 1 |  |
| Postictal state |  | 1 |  |
| Tonsillar erythema |  | 1 |  |
| Malignant lymphoid neoplasm |  | 1 |  |
| Lip dry |  | 3 |  |
| Lip haemorrhage |  | 1 |  |
| Liver injury |  | 1 |  |
| Localised infection |  | 1 |  |
| Lower limb fracture |  | 1 |  |
| Lung consolidation |  | 1 |  |
| Lung disorder |  | 3 |  |
| Lung neoplasm |  | 1 |  |
| Lupus nephritis |  | 1 |  |
| Metabolic dysfunction-associated steatohepatitis |  | 1 |  |
| Lymphoid hyperplasia of intestine |  | 1 |  |
| Lid sulcus deepened |  | 2 |  |
| Maternal exposure during pregnancy |  | 1 |  |
| Mean cell haemoglobin concentration increased |  | 1 |  |
| Medulloblastoma |  | 1 |  |
| Melaena |  | 2 |  |
| Meningitis |  | 1 |  |
| Meningitis bacterial |  | 1 |  |
| Meningitis tuberculous |  | 3 |  |
| Menstrual disorder |  | 1 |  |
| Mental disorder |  | 1 |  |
| Pregnancy |  | 1 |  |
| Lymph node tuberculosis |  | 1 |  |
| Internal haemorrhage |  | 91 |  |
| Zika virus infection |  | 2 |  |
| Hyperthyroidism |  | 1 |  |
| Hypertrophy |  | 9 |  |
| Hyperuricaemia |  | 1 |  |
| Hypoglycaemia |  | 1 |  |
| Hypomenorrhoea |  | 1 |  |
| Hyposmia |  | 1 |  |
| Hypoxia |  | 4 |  |
| Immobile |  | 2 |  |
| Immunodeficiency |  | 2 |  |
| Limb deformity |  | 2 |  |
| Injury |  | 1 |  |
| Ligament sprain |  | 2 |  |
| Intestinal obstruction |  | 2 |  |
| Intestinal perforation |  | 1 |  |
| Intracranial mass |  | 3 |  |
| Irregular breathing |  | 1 |  |
| Kawasaki's disease |  | 1 |  |
| Kidney enlargement |  | 1 |  |
| Lacrimation increased |  | 3 |  |
| Leptospirosis |  | 1 |  |
| Leukaemia |  | 14 |  |
| Moaning |  | 2 |  |
| Impulsive behaviour |  | 1 |  |
| Pericarditis |  | 1 |  |
| Obstructive airways disorder |  | 1 |  |
| Ocular icterus |  | 1 |  |
| Oral candidiasis |  | 1 |  |
| Oral herpes |  | 1 |  |
| Oral pain |  | 1 |  |
| Orthopnoea |  | 2 |  |
| Osteosarcoma |  | 1 |  |
| Ovarian cancer stage IV |  | 1 |  |
| Pancreatic disorder |  | 1 |  |
| Metabolic acidosis |  | 1 |  |
| Parosmia |  | 1 |  |
| Neuromyopathy |  | 1 |  |
| Periorbital inflammation |  | 1 |  |
| Peritonitis |  | 1 |  |
| Pharyngotonsillitis |  | 3 |  |
| Physical disability |  | 1 |  |
| Pneumonitis |  | 2 |  |
| Pneumothorax |  | 3 |  |
| Polydipsia |  | 3 |  |
| Polymenorrhoea |  | 1 |  |
| Portal tract inflammation |  | 1 |  |
| Post procedural complication |  | 1 |  |
| Pancreatic enlargement |  | 2 |  |
| Myocardial infarction |  | 1 |  |
| Mood swings |  | 1 |  |
| Motion sickness |  | 1 |  |
| Mouth haemorrhage |  | 10 |  |
| Mouth injury |  | 1 |  |
| Multi-organ disorder |  | 16 |  |
| Multiple injuries |  | 1 |  |
| Multiple organ dysfunction syndrome |  | 24 |  |
| Mumps |  | 2 |  |
| Muscle strain |  | 1 |  |
| Mutism |  | 1 |  |
| Noninfective encephalitis |  | 57 |  |
| Myocardial haemorrhage |  | 11 |  |
| Neutrophil count increased |  | 1 |  |
| Nail discolouration |  | 3 |  |
| Near drowning |  | 1 |  |
| Neck mass |  | 3 |  |
| Neoplasm malignant |  | 2 |  |
| Neoplasm progression |  | 1 |  |
| Nephrotic syndrome |  | 2 |  |
| Nervous system disorder |  | 15 |  |
| Neurogenic shock |  | 1 |  |
| Neurological infection |  | 4 |  |
| Hyperplasia |  | 1 |  |
| Myeloid leukaemia |  | 2 |  |
